# Temporal Refinements for Guarded Recursive Types

**DOI:** 10.1007/978-3-030-72019-3_20

**Published:** 2021-03-23

**Authors:** Guilhem Jaber, Colin Riba

**Affiliations:** Imperial College, London, UK; 9 Université de Nantes, LS2N CNRS, Inria, Nantes, France; 10 Univ Lyon, EnsL, UCBL, CNRS, LIP, F-69342 Lyon Cedex 07, France

**Keywords:** coinductive types, guarded recursive types, $\mu$-calculus, refinement types, topos of trees

## Abstract

We propose a logic for temporal properties of higher-order programs that handle infinite objects like streams or infinite trees, represented via coinductive types. Specifications of programs use safety and liveness properties. Programs can then be proven to satisfy their specification in a compositional way, our logic being based on a type system.

The logic is presented as a refinement type system over the guarded $\lambda$-calculus, a $\lambda$-calculus with guarded recursive types. The refinements are formulae of a modal $\mu$-calculus which embeds usual temporal modal logics such as LTL and CTL. The semantics of our system is given within a rich structure, the topos of trees, in which we build a realizability model of the temporal refinement type system.

### 1 Introduction

Functional programming is by now well established to handle infinite data, thanks to declarative definitions and equational reasoning on high-level abstractions, in particular when infinite objects are represented with coinductive types. In such settings, programs in general do not terminate, but are expected to compute a part of their output in finite time. For example, a program expected to generate a stream should produce the next element in finite time: it is *productive*.

Our goal is to prove input-output temporal properties of higher-order programs that handle coinductive types. Logics like $\mathsf{LTL}$, $\mathsf{CTL}$ or the modal $\mu$-calculus are widely used to formulate, on infinite objects, safety and liveness properties. Safety properties state that some "bad" event will not occur, while liveness properties specify that "something good" will happen (see e.g. [9]). Typically, modalities like $\Box$ (*always*) or $\Diamond$ (*eventually*) are used to write properties of streams or infinite trees and specifications of programs over such data.

We consider temporal refinement types $\{A \mid \varphi\}$, where $A$ is a standard type of our programming language, and $\varphi$ is a formula of the modal $\mu$-calculus. Using

* This work was partially supported by the ANR-14-CE25-0007 - RAPIDO and by the LABEX MILYON (ANR-10-LABX-0070) of Université de Lyon.

refinement types [22], temporal connectives are not reflected in the programming language, and programs are formally independent from the shape of their temporal specifications. One can thus give different refinement types to the same program. For example, the following two types can be given to the same map function on streams:

$$\begin{array}{l}\mathsf{map} : (\{B \mid \psi\} \to \{A \mid \varphi\}) \longrightarrow \{\mathsf{Str}\,B \mid \Box\Diamond[\mathsf{hd}]\psi\} \longrightarrow \{\mathsf{Str}\,A \mid \Box\Diamond[\mathsf{hd}]\varphi\} \\ \mathsf{map} : (\{B \mid \psi\} \to \{A \mid \varphi\}) \longrightarrow \{\mathsf{Str}\,B \mid \Diamond\Box[\mathsf{hd}]\psi\} \longrightarrow \{\mathsf{Str}\,A \mid \Diamond\Box[\mathsf{hd}]\varphi\}\end{array} \quad (\star)$$

These types mean that given $f : B \to A$ s.t. $f(b)$ satisfies $\varphi$ if $b$ satisfies $\psi$, the function $(\mathsf{map}\ f)$ takes a stream with infinitely many (resp. ultimately all) elements satisfying $\psi$ to one with infinitely many (resp. ultimately all) elements satisfying $\varphi$. For $\varphi$ a formula over $A$, $[\mathsf{hd}]\varphi$ is a formula over streams of $A$'s which holds on a given stream if $\varphi$ holds on its head element.

It is undecidable whether a given higher-order program satisfies a given input-output temporal property written with formulae of the modal $\mu$-calculus [41]. Having a type system is a partial workaround to this obstacle, which moreover enables to reason compositionally on programs, by decomposing a specification to the various components of a program in order to prove its global specification.

Our system is built on top of the guarded $\lambda$-calculus [18], a higher-order programming language with guarded recursion [52]. Guarded recursion is a simple device to control and reason about unfoldings of fixpoints. It can represent coinductive types [50] and provides a syntactic compositional productivity check [5].

Safety properties (e.g. $\Box[\mathsf{hd}]\varphi$) can be correctly represented with guarded fixpoints, but not liveness properties (e.g. $\Diamond[\mathsf{hd}]\varphi$, $\Diamond\Box[\mathsf{hd}]\varphi$, $\Box\Diamond[\mathsf{hd}]\varphi$). Combining liveness with guarded recursion is a challenging problem since guarded fixpoints tend to have unique solutions. Existing approaches to handle temporal types in presence of guarded recursion face similar difficulties. Functional reactive programming (FRP) [21] provides a Curry-Howard correspondence for temporal logics [32,33,17] in which logical connectives are reflected as programming constructs. When combining FRP with guarded recursion [44,7], and in particular to handle liveness properties [8], uniqueness of guarded fixpoints is tempered by specific recursors for temporal types.

Our approach is different from [8], as we wish as much as possible the logical level not to impact the program level. We propose a two level system, with the lower or *internal* level, which interacts with guarded recursion and at which only safety properties are correctly represented, and the higher or *external* one, at which liveness properties are correctly handled, but without direct access to guarded recursion. By restricting to the alternation-free modal $\mu$-calculus, in which fixpoints can always be computed in $\omega$-steps, one can syntactically reason on finite unfoldings of liveness properties, thus allowing for crossing down the safety barrier. Soundness is proved by a realizability interpretation based on the semantics of guarded recursion in the topos of trees [13], which correctly represents the usual set-theoretic final coalgebras of polynomial coinductive types [50].

We provide example programs involving linear structures (colists, streams, fair streams [17,8]) and branching structures (resumptions *à la* [44]), for which

$$\begin{array}{rll} \mathsf{Cons}^{\mathsf{g}} & := \lambda x.\lambda s.\,\mathsf{fold}(\langle x, s\rangle) : A \to \blacktriangleright \mathsf{Str}^{\mathsf{g}}\,A \to \mathsf{Str}^{\mathsf{g}}\,A \\ \mathsf{hd}^{\mathsf{g}} & := \lambda s.\pi_0(\mathsf{unfold}\ s) & : \mathsf{Str}^{\mathsf{g}}\,A \to A \\ \mathsf{tl}^{\mathsf{g}} & := \lambda s.\pi_1(\mathsf{unfold}\ s) & : \mathsf{Str}^{\mathsf{g}}\,A \to \blacktriangleright \mathsf{Str}^{\mathsf{g}}\,A \\ \mathsf{map}^{\mathsf{g}} & := \lambda f.\mathsf{fix}(g).\lambda s.\,\mathsf{Cons}^{\mathsf{g}}\ (f(\mathsf{hd}^{\mathsf{g}}\ s))\ (g \circledast (\mathsf{tl}^{\mathsf{g}}\ s)) & : (B \to A) \to \mathsf{Str}^{\mathsf{g}}\,B \to \mathsf{Str}^{\mathsf{g}}\,A \end{array}$$

**Fig. 1.** Constructor, Destructors and Map on Guarded Streams.

we prove liveness properties similar to ($\star$) above. Our system also handles safety properties on breadth-first (infinite) tree traversals *à la* [35] and [10].

**Organization of the paper.** We give an overview of our approach in §2. Then §3 presents the syntax of the guarded $\lambda$-calculus. Our base temporal logic (without liveness) is introduced in §4, and is used to define our refinement type system in §5. Liveness properties are handled in §6. The semantics is given in §7, and §8 presents examples. Finally, we discuss related work in §9 and future work in §10. Table 4 (§8) gathers the main refinement types we can give to example functions, most of them defined in Table 3. Omitted material is available in [28].

### 2 Outline

**Overview of the Guarded $\lambda$-Calculus.** Guarded recursion enforces productivity of programs using a type system equipped with a type modality $\blacktriangleright$, in order to indicate that one has access to a value not right now but only "later". One can define guarded streams $\mathsf{Str}^{\mathsf{g}}\,A$ over a type $A$ via the guarded recursive definition $\mathsf{Str}^{\mathsf{g}}\,A = A \times \blacktriangleright \mathsf{Str}^{\mathsf{g}}\,A$. Streams that inhabit this type have their head available now, but their tail only one step in the future. The type modality $\blacktriangleright$ is reflected in programs with the $\mathsf{next}$ operation. One also has a fixpoint constructor on terms $\mathsf{fix}(x).M$ for guarded recursive definitions. They are typed with

$$\frac{\mathcal{E} \vdash M : A}{\mathcal{E} \vdash \mathsf{next}(M) : \blacktriangleright A} \qquad \frac{\mathcal{E}, x : \blacktriangleright A \vdash M : A}{\mathcal{E} \vdash \mathsf{fix}(x).M : A}$$

This allows for the constructor and basic destructors on guarded streams to be defined as in Fig. 1, where $\mathsf{fold}(-)$ and $\mathsf{unfold}(-)$ are explicit operations for folding and unfolding guarded recursive types. In the following, we use the infix notation $a ::^{\mathsf{g}} s$ for $\mathsf{Cons}^{\mathsf{g}}\ a\ s$. Using the fact that the type modality $\blacktriangleright$ is an applicative functor [49], we can distribute $\blacktriangleright$ over the arrow type. This is represented in the programming language by the infix applicative operator $\circledast$. With it, one can define the usual map function on guarded streams as in Fig. 1.

**Compositional Safety Reasoning on Streams.** Given a property $\varphi$ on a type $A$, we would like to consider a subtype of $\mathsf{Str}^{\mathsf{g}}\,A$ that selects those streams whose elements all satisfy $\varphi$. To do so, we use a temporal modal formula $\Box[\mathsf{hd}]\varphi$,

| Typed Formulae | Provability | Refinement Types | Subtyping | Typing |
|---|---|---|---|---|
| $\Sigma \vdash \varphi : A$ | $\vdash^A \varphi$ | $\{A \mid \varphi\}$ | $T \leq U$ | $\mathcal{E} \vdash M : T$ |
| (§4) | (where $\vdash \varphi : A$, §4) | (where $\vdash \varphi : A$, §5) | ($T$, $U$ refinement types, §5) | |

**Table 1.** Syntactic Classes and Judgments.

and consider the *refinement type* $\{\mathsf{Str}^{\mathsf{g}}\, A \mid \Box[\mathsf{hd}]\varphi\}$. Suppose for now that we can give the following refinement types to the basic stream operations:

$$\begin{aligned} \mathsf{hd}^{\mathsf{g}} &: \{\mathsf{Str}^{\mathsf{g}}\, A \mid \Box[\mathsf{hd}]\varphi\} \longrightarrow \{A \mid \varphi\} \\ \mathsf{tl}^{\mathsf{g}} &: \{\mathsf{Str}^{\mathsf{g}}\, A \mid \Box[\mathsf{hd}]\varphi\} \longrightarrow \blacktriangleright \{\mathsf{Str}^{\mathsf{g}}\, A \mid \Box[\mathsf{hd}]\varphi\} \\ \mathsf{Cons}^{\mathsf{g}} &: \{A \mid \varphi\} \longrightarrow \blacktriangleright \{\mathsf{Str}^{\mathsf{g}}\, A \mid \Box[\mathsf{hd}]\varphi\} \longrightarrow \{\mathsf{Str}^{\mathsf{g}}\, A \mid \Box[\mathsf{hd}]\varphi\} \end{aligned}$$

By using the standard typing rules for $\lambda$-abstraction and application, together with the rules to type $\mathsf{fix}(x).M$ and $\circledast$, we can type the function $\mathsf{map}^{\mathsf{g}}$ as

$$\mathsf{map}^{\mathsf{g}} : (\{B \mid \psi\} \to \{A \mid \varphi\}) \longrightarrow \{\mathsf{Str}^{\mathsf{g}}\, B \mid \Box[\mathsf{hd}]\psi\} \longrightarrow \{\mathsf{Str}^{\mathsf{g}}\, A \mid \Box[\mathsf{hd}]\varphi\}$$

**A Manysorted Temporal Logic.** Our logical language, taken with minor adaptations from [30], is *manysorted*: for each type $A$ we have formulae *of type* $A$ (notation $\vdash \varphi : A$), where $\varphi$ selects inhabitants of $A$.

We use atomic modalities ($[\pi_i], [\mathsf{fold}], [\mathsf{next}], \dots$) in refinements to navigate between types (see Fig. 5, §4). For instance, a formula $\varphi$ of type $A_0$, specifying a property over the inhabitants of $A_0$, can be lifted to the formula $[\pi_0]\varphi$ of type $A_0 \times A_1$, which intuitively describes those inhabitants of $A_0 \times A_1$ whose first component satisfy $\varphi$. Given a formula $\varphi$ of type $A$, one can define its “head lift” $[\mathsf{hd}]\varphi$ of type $\mathsf{Str}^{\mathsf{g}}\, A$, that enforces $\varphi$ to be satisfied on the head of the provided stream. Also, one can define a modality $\bigcirc$ such that given a formula $\psi : \mathsf{Str}^{\mathsf{g}}\, A$, the formula $\bigcirc\psi : \mathsf{Str}^{\mathsf{g}}\, A$ enforces $\psi$ to be satisfied on the tail of the provided stream. These modalities are obtained resp. as $[\mathsf{hd}]\varphi := [\mathsf{fold}][\pi_0]\varphi$ and $\bigcirc\varphi := [\mathsf{fold}][\pi_1][\mathsf{next}]\varphi$. We similarly have atomic modalities $[\mathsf{in}_0], [\mathsf{in}_1]$ on sum types. For instance, on the type of guarded colists defined as $\mathsf{CoList}^{\mathsf{g}}\, A := \mathsf{Fix}(X).\ \mathbf{1} + A \times \blacktriangleright X$, we can express the fact that a colist is empty (resp. non-empty) with the formula $[\mathsf{nil}] := [\mathsf{fold}][\mathsf{in}_0]\top$ (resp. $[\neg\mathsf{nil}] := [\mathsf{fold}][\mathsf{in}_1]\top$).

We also provide a deduction system $\vdash^A \varphi$ on temporal modal formulae. This deduction system is used to define a subtyping relation $T \leq U$ between refinement types, with $\{A \mid \varphi\} \leq \{A \mid \psi\}$ when $\vdash^A \varphi \Rightarrow \psi$. The subtyping relation thus incorporates logical reasoning in the type system.

In addition, we have greatest fixpoints formulae $\nu\alpha\varphi$ (so that formulae can have free typed propositional variables), equipped with Kozen's reasoning principles [43]. In particular, we can form an *always* modality as $\Box\varphi := \nu\alpha.\ \varphi \wedge \bigcirc\alpha$, with $\Box\varphi : \mathsf{Str}^{\mathsf{g}}\, A$ if $\varphi : \mathsf{Str}^{\mathsf{g}}\, A$. The formula $\Box\varphi$ holds on a stream $s = (s_i \mid i \geq 0)$, iff $\varphi$ holds on every substream $(s_i \mid i \geq n)$ for $n \geq 0$. If we rather start with $\psi : A$, one first need to lift it to $[\mathsf{hd}]\psi : \mathsf{Str}^{\mathsf{g}}\, A$. Then $\Box[\mathsf{hd}]\psi$ means that all the elements of the stream satisfies $\psi$, since all its suffixes satisfy $[\mathsf{hd}]\psi$.

Table 1 summarizes the different judgments used in this paper.

**Beyond Safety.** In order to handle liveness properties, we also need to have least fixpoints formulae $\mu\alpha\varphi$. For example, this would give the *eventually* modality $\Diamond\varphi := \mu\alpha.\ \varphi \vee \bigcirc\alpha$. With Kozen-style rules, one could then give the following two types to the guarded stream constructor:

$$\begin{aligned}\mathsf{Cons}^{\mathsf{g}} &: \{A \mid \varphi\} \longrightarrow \blacktriangleright \mathsf{Str}^{\mathsf{g}}\, A \longrightarrow \{\mathsf{Str}^{\mathsf{g}}\, A \mid \Diamond[\mathsf{hd}]\varphi\}\\ \mathsf{Cons}^{\mathsf{g}} &: A \longrightarrow \blacktriangleright \{\mathsf{Str}^{\mathsf{g}}\, A \mid \Diamond[\mathsf{hd}]\varphi\} \longrightarrow \{\mathsf{Str}^{\mathsf{g}}\, A \mid \Diamond[\mathsf{hd}]\varphi\}\end{aligned}$$

But consider a finite base type $\mathtt{B}$ with two distinguished elements $\mathtt{a}, \mathtt{b}$, and suppose that we have access to a modality $[\mathtt{b}]$ on $\mathtt{B}$ so that terms inhabiting $\{\mathtt{B} \mid [\mathtt{b}]\}$ must be equal to $\mathtt{b}$. Using the above types for $\mathsf{Cons}^{\mathsf{g}}$, we could type the stream with constant value $\mathtt{a}$, defined as $\mathsf{fix}(s).\mathtt{a} ::^{\mathsf{g}} s$, with the type $\{\mathsf{Str}^{\mathsf{g}}\, \mathtt{B} \mid \Diamond[\mathsf{hd}][\mathtt{b}]\}$ that is supposed to enforce the existence of an occurrence of $\mathtt{b}$ in the stream. Similarly, on colists we would have $\mathsf{fix}(s).\mathtt{a} ::^{\mathsf{g}} s$ of type $\{\mathsf{CoList}^{\mathsf{g}}\, \mathtt{B} \mid \Diamond[\mathsf{nil}]\}$, while $\Diamond[\mathsf{nil}]$ expresses that a colist will eventually contain a nil, and is thus finite. Hence, liveness properties may interact quite badly with guarded recursion. Let us look at this in a semantic model of guarded recursion.

***Internal*** **Semantics in the Topos of Trees.** The types of the guarded $\lambda$-calculus can be interpreted as sequences of sets $(X(n))_{n>0}$ where $X(n)$ represents the values available "at time $n$". In order to interpret guarded recursion, one also needs to have access to functions $r^X_n : X(n+1) \to X(n)$, which tell how values "at $n{+}1$" can be restricted (actually most often truncated) to values "at $n$". This means that the objects used to represent types are in fact *presheaves* over the poset $(\mathbb{N} \setminus \{0\}, \leq)$. The category $\mathcal{S}$ of such presheaves is the *topos of trees* [13]. For instance, the type $\mathsf{Str}^{\mathsf{g}}\, \mathtt{B}$ of guarded streams over a finite base type $\mathtt{B}$ is interpreted in $\mathcal{S}$ as $(\mathtt{B}^n)_{n>0}$ , with restriction maps taking $(\mathtt{b}_0, \ldots, \mathtt{b}_{n-1}, \mathtt{b}_n)$ to $(\mathtt{b}_0, \ldots, \mathtt{b}_{n-1})$. We write $[\![A]\!]$ for the interpretation of a type $A$ in $\mathcal{S}$.

**The Necessity of an** ***External*** **Semantics.** The topos of trees cannot correctly handle liveness properties. For instance, the formula $\Diamond[\mathsf{hd}][\mathtt{b}]$ cannot describe in $\mathcal{S}$ the set of streams that contain at least one occurrence of $\mathtt{b}$. Indeed, the interpretation of $\Diamond[\mathsf{hd}][\mathtt{b}]$ in $\mathcal{S}$ is a sequence $(C(n))_{n>0}$ with $C(n) \subseteq \mathtt{B}^n$. But any element of $\mathtt{B}^n$ can be extended to a stream which contains an occurrence of $\mathtt{b}$. Hence $C(n)$ should be equal to $\mathtt{B}^n$, and the interpretation of $\Diamond[\mathsf{hd}][\mathtt{b}]$ is the whole $[\![\mathsf{Str}^{\mathsf{g}}\, \mathtt{B}]\!]$. More generally, guarded fixpoints have unique solutions in the topos of trees [13], and $\Diamond\varphi = \mu\alpha.\ \varphi \vee \bigcirc\varphi$ gets the same interpretation as $\nu\alpha.\ \varphi \vee \bigcirc\alpha$.

We thus have a formal system with least and greatest fixpoints, that has a semantics inside the topos of trees, but which does not correctly handle least fixpoints. On the other hand, it was shown by [50] that the interpretation of guarded polynomial (*i.e.* first-order) recursive types in $\mathcal{S}$ induces final coalgebras for the corresponding polynomial functors on the category **Set** of usual sets and functions. This applies e.g. to streams and colists. Hence, it makes sense to think of interpreting least fixpoint formulae over such types *externally*, in **Set**.

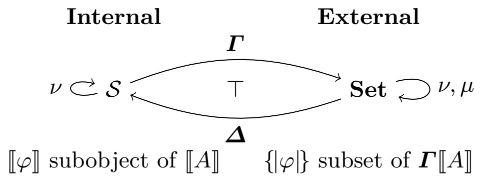

$$\begin{aligned} [\![\blacksquare A]\!] &:= \boldsymbol{\Delta\Gamma}[\![A]\!] \\ [\![[\mathsf{box}]\varphi]\!] &:= \boldsymbol{\Delta}\,\{\!|\varphi|\!\} \quad (\varphi : A,\ [\mathsf{box}]\varphi : \blacksquare A) \\ \{\!|\varphi|\!\} &= \boldsymbol{\Gamma}[\![\varphi]\!] \quad \text{(if } \varphi \text{ is safe)} \end{aligned}$$

**Fig. 2.** Internal and External Semantics

**The Constant Type Modality.** Figure 2 represents adjoint functors $\boldsymbol{\Gamma} : \mathcal{S} \to \mathbf{Set}$ and $\boldsymbol{\Delta} : \mathbf{Set} \to \mathcal{S}$. To correctly handle least fixpoints $\mu\alpha\varphi : A$, we would like to see them as subsets of $\boldsymbol{\Gamma}[\![A]\!]$ in **Set** rather than subobjects of $[\![A]\!]$ in $\mathcal{S}$. On the other hand, the internal semantics in $\mathcal{S}$ is still necessary to handle definitions by guarded recursion. We navigate between the internal semantics in $\mathcal{S}$ and the external semantics in **Set** via the adjunction $\boldsymbol{\Delta} \dashv \boldsymbol{\Gamma}$. This adjunction induces a comonad $\boldsymbol{\Delta\Gamma}$ on $\mathcal{S}$, which is represented in the guarded $\lambda$-calculus of [18] by the *constant* type modality $\blacksquare$. This gives *coinductive* versions of guarded recursive types, e.g. $\mathsf{Str}\,A := \blacksquare\,\mathsf{Str}^{\mathsf{g}}\,A$ for streams and $\mathsf{CoList}\,A := \blacksquare\,\mathsf{CoList}^{\mathsf{g}}\,A$ for colists, which allow for productive but not causal programs [18, Ex. 1.10.(3)].

Each formula gets two interpretations: $[\![\varphi]\!]$ in $\mathcal{S}$ and $\{\!|\varphi|\!\}$ in **Set**. The external semantics $\{\!|\varphi|\!\}$ handles least fixpoints in the standard set-theoretic way, thus the two interpretations differ in general. But we do have $\{\!|\varphi|\!\} = \boldsymbol{\Gamma}[\![\varphi]\!]$ when $\varphi$ is *safe* (Def. 6.5), that is, when $\varphi$ describes a safety property. We have a modality $[\mathsf{box}]\varphi$ which lifts $\varphi : A$ to $\blacksquare A$. By defining $[\![[\mathsf{box}]\varphi]\!] := \boldsymbol{\Delta}\,\{\!|\varphi|\!\}$, we correctly handle the least fixpoints which are guarded by a $[\mathsf{box}]$ modality. When $\varphi$ is safe, we can navigate between $\{\blacksquare A \mid [\mathsf{box}]\varphi\}$ and $\blacksquare\,\{A \mid \varphi\}$, thus making available the comonad structure of $\blacksquare$ on $[\mathsf{box}]\varphi$. Note that $[\mathsf{box}]$ is unrelated to $\Box$.

**Approximating Least Fixpoints.** For proving liveness properties on functions defined by guarded recursion, one needs to navigate between e.g. $[\mathsf{box}]\Diamond\varphi$ and $\Diamond\varphi$, while $\Diamond\varphi$ is in general unsafe. The fixpoint $\Diamond\varphi = \mu\alpha.\varphi \vee \bigcirc\alpha$ is *alternation-free* (see e.g. [16, §4.1]). This implies that $\Diamond\varphi$ can be seen as the supremum of the $\bigcirc^m\varphi$ for $m \in \mathbb{N}$, where each $\bigcirc^m\varphi$ is safe when $\varphi$ is safe. More generally, we can approximate alternation-free $\mu\alpha\varphi$ by their finite unfoldings $\varphi^m(\bot)$, *à la* Kleene. We extend the logic with finite iterations $\mu^k\alpha\varphi$, where $k$ is an *iteration variable*, and where $\mu^k\alpha\varphi$ is seen as $\varphi^k(\bot)$. Let $\Diamond^k\varphi := \mu^k\alpha.\ \varphi\vee\bigcirc\alpha$. If $\varphi$ is safe then so is $\Diamond^k\varphi$. For safe $\varphi$, $\psi$, we have the following refinement typings for the guarded recursive $\mathsf{map}^{\mathsf{g}}$ and its coinductive lift $\mathsf{map}$:

$$\begin{aligned} \mathsf{map}^{\mathsf{g}} &: (\{B \mid \psi\} \to \{A \mid \varphi\}) \to \left\{\mathsf{Str}^{\mathsf{g}}\,B \;\middle|\; \Diamond^k[\mathsf{hd}]\psi\right\} \quad \to \left\{\mathsf{Str}^{\mathsf{g}}\,A \;\middle|\; \Diamond^k[\mathsf{hd}]\varphi\right\} \\ \mathsf{map} &: (\{B \mid \psi\} \to \{A \mid \varphi\}) \to \{\mathsf{Str}\,B \mid [\mathsf{box}]\Diamond[\mathsf{hd}]\psi\} \to \{\mathsf{Str}\,A \mid [\mathsf{box}]\Diamond[\mathsf{hd}]\varphi\} \end{aligned}$$

### 3 The Pure Calculus

Our system lies on top of the guarded $\lambda$-calculus of [18]. We briefly review it here. We consider values and terms from the grammar given in Fig. 3 (left). In

$$\begin{array}{llll}
v & ::= & M,N ::= & v \mid x & E ::= \bullet \\
\mid & \lambda x.M & \mid & MN & \mid EM \\
\mid & \langle M_0, M_1\rangle & \mid & \pi_0(M) & \mid \pi_0(E) \\
\mid & \langle\rangle & \mid & \pi_1(M) & \mid \pi_1(E) \\
\mid & \mathsf{in}_0(M) & \mid & \mathsf{case}\, M \,\mathsf{of}\, (x.M_0|x.M_1) & \mid \mathsf{case}\, E \,\mathsf{of}\, (x.M_0|x.M_1) \\
\mid & \mathsf{in}_1(M) & & & \\
\mid & \mathsf{fold}(M) & \mid & \mathsf{unfold}(M) & \mid \mathsf{unfold}(E) \\
\mid & \mathsf{box}_\sigma(M) & \mid & \mathsf{unbox}(M) & \mid \mathsf{unbox}(E) \\
\mid & \mathsf{next}(M) & \mid & \mathsf{prev}_\sigma(M) & \mid \mathsf{prev}_{[]}(E) \\
 & & \mid & M \circledast N & \mid E \circledast M \\
 & & \mid & \mathsf{fix}(x).M & \mid v \circledast E
\end{array}$$

$$\begin{array}{rcl}
(\lambda x.M)N & \rightsquigarrow & M[N/x] \\
\pi_i(\langle M_0, M_1\rangle) & \rightsquigarrow & M_i \\
\mathsf{case}\,\mathsf{in}_i(M)\,\mathsf{of}\,(x.N_0|x.N_1) & \rightsquigarrow & N_i[M/x] \\
\mathsf{unfold}(\mathsf{fold}(M)) & \rightsquigarrow & M \\
\mathsf{fix}(x).M & \rightsquigarrow & M[\mathsf{next}(\mathsf{fix}(x).M)/x] \\
\mathsf{next}(M) \circledast \mathsf{next}(N) & \rightsquigarrow & \mathsf{next}(MN) \\
\mathsf{unbox}(\mathsf{box}_\sigma(M)) & \rightsquigarrow & M\sigma \\
\mathsf{prev}_{[]}(\mathsf{next}(M)) & \rightsquigarrow & M \\
\mathsf{prev}_\sigma(M) & \rightsquigarrow & \mathsf{prev}_{[]}(M\sigma) \quad (\sigma \neq [])
\end{array}$$

$$\frac{M \rightsquigarrow N}{E[M] \rightsquigarrow E[N]}$$

**Fig. 3.** Syntax and Operational Semantics of the Pure Calculus.

both $\mathsf{box}_\sigma(M)$ and $\mathsf{prev}_\sigma(M)$, $\sigma$ is a *delayed substitution* of the form $\sigma = [x_1 \mapsto M_1, \ldots, x_k \mapsto M_k]$ and such that $\mathsf{box}_\sigma(M)$ and $\mathsf{prev}_\sigma(M)$ bind $x_1, \ldots, x_k$ in $M$. We use the following conventions of [18]: $\mathsf{box}(M)$ and $\mathsf{prev}(M)$ (without indicated substitution) stand resp. for $\mathsf{box}_{[]}(M)$ and $\mathsf{prev}_{[]}(M)$ *i.e.* bind no variable of $M$. Moreover, $\mathsf{box}_\iota(M)$ stands for $\mathsf{box}_{[x_1 \mapsto x_1, \ldots, x_k \mapsto x_k]}(M)$ where $x_1, \ldots, x_k$ is a list of all free variables of $M$, and similarly for $\mathsf{prev}_\iota(M)$. We consider the weak call-by-name reduction of [18], recalled in Fig. 3 (right).

*Pure types* (notation $A, B,$ etc.) are the closed types over the grammar

$$A \;::=\; \mathbf{1} \mid A + A \mid A \times A \mid A \to A \mid \blacktriangleright A \mid X \mid \mathsf{Fix}(X).A \mid \blacksquare A$$

where, (1) in the case $\mathsf{Fix}(X).A$, each occurrence of $X$ in $A$ must be guarded by a $\blacktriangleright$, and (2) in the case of $\blacksquare A$, the type $A$ is closed (*i.e.* has no free type variable). Guarded recursive types are built with the fixpoint constructor $\mathsf{Fix}(X).A$, which allows for $X$ to appear in $A$ both at positive and negative positions, but only under a $\blacktriangleright$. In this paper we shall only consider positive types.

*Example 3.1.* We can code a finite base type $\mathtt{B} = \{\mathtt{b}_1, \ldots, \mathtt{b}_n\}$ as a sum of unit types $\sum_{i=1}^n \mathbf{1} = \mathbf{1} + (\cdots + \mathbf{1})$, where the $i$th component of the sum is intended to represent the element $\mathtt{b}_i$ of $\mathtt{B}$. At the term level, the elements of $\mathtt{B}$ are represented as compositions of injections $\mathsf{in}_{j_1}(\mathsf{in}_{j_2}(\ldots \mathsf{in}_{j_i}\langle\rangle))$. For instance, Booleans are represented by $\mathsf{Bool} := \mathbf{1} + \mathbf{1}$, with $\mathsf{tt} := \mathsf{in}_0(\langle\rangle)$ and $\mathsf{ff} := \mathsf{in}_1(\langle\rangle)$.

*Example 3.2.* Besides streams ($\mathsf{Str}^{\mathsf{g}} A$), colists ($\mathsf{CoList}^{\mathsf{g}} A$), conatural numbers ($\mathsf{CoNat}^{\mathsf{g}}$) and infinite binary trees ($\mathsf{Tree}^{\mathsf{g}} A$), we consider a type $\mathsf{Res}^{\mathsf{g}} A$ of *resumptions* (parametrized by $\mathtt{I}, \mathtt{O}$) adapted from [44], and a higher-order recursive type $\mathsf{Rou}^{\mathsf{g}} A$, used in Martin Hofmann's breadth-first tree traversal (see e.g. [10]):

$$\begin{array}{rlrl}
\mathsf{Tree}^{\mathsf{g}} A := & \mathsf{Fix}(X).\ A \times (\blacktriangleright X \times \blacktriangleright X) & \mathsf{CoNat}^{\mathsf{g}} := & \mathsf{Fix}(X).\ \mathbf{1} + \blacktriangleright X \\
\mathsf{Res}^{\mathsf{g}} A := & \mathsf{Fix}(X).\ A + (\mathtt{I} \to (\mathtt{O} \times \blacktriangleright X)) & \mathsf{Rou}^{\mathsf{g}} A := & \mathsf{Fix}(X).\ \mathbf{1} + ((\blacktriangleright X \to \blacktriangleright A) \to A)
\end{array}$$

Some typing rules of the pure calculus are given in Fig. 4, where a pure type $A$ is *constant* if each occurrence of $\blacktriangleright$ in $A$ is guarded by a $\blacksquare$. The omitted rules are the standard ones for simple types with finite sums and products [28, §A].

$$\frac{\mathcal{E} \vdash M : A[\mathsf{Fix}(X).A/X]}{\mathcal{E} \vdash \mathsf{fold}(M) : \mathsf{Fix}(X).A} \qquad \frac{\mathcal{E} \vdash M : \mathsf{Fix}(X).A}{\mathcal{E} \vdash \mathsf{unfold}(M) : A[\mathsf{Fix}(X).A/X]} \qquad \frac{\mathcal{E} \vdash M : \blacktriangleright(B \to A) \qquad \mathcal{E} \vdash N : \blacktriangleright B}{\mathcal{E} \vdash M \circledast N : \blacktriangleright A}$$

$$\frac{\mathcal{E} \vdash M : A}{\mathcal{E} \vdash \mathsf{next}(M) : \blacktriangleright A} \qquad \frac{x_1 : A_1, \dots, x_k : A_k \vdash M : \blacktriangleright A \qquad \mathcal{E} \vdash M_i : A_i \text{ with } A_i \text{ constant for } 1 \leq i \leq k}{\mathcal{E} \vdash \mathsf{prev}_{[x_1 \mapsto M_1, \dots, x_k \mapsto M_k]}(M) : A}$$

$$\frac{x_1 : A_1, \dots, x_k : A_k \vdash M : A \qquad \mathcal{E} \vdash M_i : A_i \text{ with } A_i \text{ constant for } 1 \leq i \leq k}{\mathcal{E} \vdash \mathsf{box}_{[x_1 \mapsto M_1, \dots, x_k \mapsto M_k]}(M) : \blacksquare A} \qquad \frac{\mathcal{E} \vdash M : \blacksquare A}{\mathcal{E} \vdash \mathsf{unbox}(M) : A}$$

**Fig. 4.** Typing Rules of the Pure Calculus (excerpt).

*Example 3.3.* Figure 1 defines some operations on guarded streams. On other types of Ex. 3.2, we have e.g. the constructors of colists $\mathsf{Nil}^{\mathsf{g}} := \mathsf{fold}(\mathsf{in}_0\langle\rangle) : \mathsf{CoList}^{\mathsf{g}}\,A$ and $\mathsf{Cons}^{\mathsf{g}} := \lambda x.\lambda xs.\mathsf{fold}(\mathsf{in}_1\langle x, xs\rangle) : A \to \blacktriangleright \mathsf{CoList}^{\mathsf{g}}\,A \to \mathsf{CoList}^{\mathsf{g}}\,A$. Infinite binary trees $\mathsf{Tree}^{\mathsf{g}}\,A$ have operations $\mathsf{son}^{\mathsf{g}}_d : \mathsf{Tree}^{\mathsf{g}}\,A \to \blacktriangleright \mathsf{Tree}^{\mathsf{g}}\,A$ for $d \in \{\ell, r\}$, $\mathsf{Node}^{\mathsf{g}} : A \to \blacktriangleright \mathsf{Tree}^{\mathsf{g}}\,A \to \blacktriangleright \mathsf{Tree}^{\mathsf{g}}\,A \to \mathsf{Tree}^{\mathsf{g}}\,A$ and $\mathsf{label}^{\mathsf{g}} : \mathsf{Tree}^{\mathsf{g}}\,A \to A$.

*Example 3.4. Coinductive* types are guarded recursive types under a $\blacksquare$. For instance $\mathsf{Str}\,A := \blacksquare\,\mathsf{Str}^{\mathsf{g}}\,A$, $\mathsf{CoList}\,A := \blacksquare\,\mathsf{CoList}^{\mathsf{g}}\,A$, $\mathsf{CoNat} := \blacksquare\,\mathsf{CoNat}^{\mathsf{g}}$ and $\mathsf{Res}\,A := \blacksquare\,\mathsf{Res}^{\mathsf{g}}\,A$, with $A$, $\mathtt{I}$, $\mathtt{O}$ *constant.* Basic operations on guarded types lift to coinductive ones. For instance

$$\begin{aligned}
\mathsf{Cons} &:= \lambda x.\lambda s.\mathsf{box}_\iota\big(\mathsf{Cons}^{\mathsf{g}}\ x\ \mathsf{next}(\mathsf{unbox}\ s)\big) &&: A \to \mathsf{Str}\,A \to \mathsf{Str}\,A \\
\mathsf{hd} &:= \lambda s.\mathsf{hd}^{\mathsf{g}}\ (\mathsf{unbox}\ s) &&: \mathsf{Str}\,A \to A \\
\mathsf{tl} &:= \lambda s.\mathsf{box}_\iota\big(\mathsf{prev}_\iota(\mathsf{tl}^{\mathsf{g}}\ (\mathsf{unbox}\ s))\big) &&: \mathsf{Str}\,A \to \mathsf{Str}\,A
\end{aligned}$$

These definitions follow a general pattern to lift a function over a guarded recursive type into one over its coinductive version, by performing an $\eta$-expansion with some $\mathsf{box}$ and $\mathsf{unbox}$ inserted in the right places. For example, one can define the map function on coinductive streams as:

$$\mathsf{map} := \lambda f.\lambda s.\mathsf{box}_\iota\big(\mathsf{map}^{\mathsf{g}}\ f\ (\mathsf{unbox}\ s)\big) : (B \to A) \longrightarrow \mathsf{Str}\,B \longrightarrow \mathsf{Str}\,A$$

## 4 A Temporal Modal Logic

We present here a logic of (modal) temporal specifications. We focus on syntactic aspects. The semantics is discussed in §7. For the moment the logic has only one form of fixpoints ($\nu\alpha\varphi$). It is extended with least fixpoints ($\mu\alpha\varphi$) in §6.

**Manysorted Modal Temporal Formulae.** The main ingredient of this paper is the logical language we use to annotate pure types when forming refinement types. This language, that we took with minor adaptations from [30], is *manysorted*: for each pure type $A$ we have formulae $\varphi$ *of type* $A$ (notation $\vdash \varphi : A$). The formulation rules of formulae are given in Fig. 5.

*Example 4.1.* Given a finite base type $\mathtt{B} = \{\mathtt{b}_1, \dots, \mathtt{b}_n\}$ as in Ex. 3.1, with element $\mathtt{b}_i$ represented by $\mathsf{in}_{j_1}(\mathsf{in}_{j_2}(\dots \mathsf{in}_{j_i}\langle\rangle))$, the formula $[\mathsf{in}_{j_1}][\mathsf{in}_{j_2}]\dots[\mathsf{in}_{j_i}]\top$ represents the singleton subset $\{\mathtt{b}_k\}$ of $\mathtt{B}$. On $\mathsf{Bool}$, we have the formulae $[\mathsf{tt}] := [\mathsf{in}_0]\top$ and $[\mathsf{ff}] := [\mathsf{in}_1]\top$ representing resp. $\mathsf{tt}$ and $\mathsf{ff}$.

$$\frac{(\alpha : A) \in \Sigma}{\Sigma \vdash \alpha : A} \qquad \frac{}{\Sigma \vdash \bot : A} \qquad \frac{}{\Sigma \vdash \top : A} \qquad \frac{\Sigma \vdash \varphi : A}{\Sigma, \alpha : B \vdash \varphi : A}$$

$$\frac{\Sigma \vdash \varphi : A \qquad \Sigma \vdash \psi : A}{\Sigma \vdash \varphi \Rightarrow \psi : A} \qquad \frac{\Sigma \vdash \varphi : A \qquad \Sigma \vdash \psi : A}{\Sigma \vdash \varphi \wedge \psi : A} \qquad \frac{\Sigma \vdash \varphi : A \qquad \Sigma \vdash \psi : A}{\Sigma \vdash \varphi \vee \psi : A}$$

$$\frac{\Sigma \vdash \varphi : A_i}{\Sigma \vdash [\pi_i]\varphi : A_0 \times A_1} \qquad \frac{\Sigma \vdash \varphi : A_i}{\Sigma \vdash [\mathsf{in}_i]\varphi : A_0 + A_1} \qquad \frac{\Sigma \vdash \psi : B \quad \Sigma \vdash \varphi : A}{\Sigma \vdash [\mathsf{ev}(\psi)]\varphi : B \to A}$$

$$\frac{\Sigma \vdash \varphi : A[\mathsf{Fix}(X).A/X]}{\Sigma \vdash [\mathsf{fold}]\varphi : \mathsf{Fix}(X).A} \qquad \frac{\Sigma \vdash \varphi : A}{\Sigma \vdash [\mathsf{next}]\varphi : \blacktriangleright A} \qquad \frac{\vdash \varphi : A}{\vdash [\mathsf{box}]\varphi : \blacksquare A}$$

$$(\nu\text{-F})\ \frac{\Sigma, \alpha : A \vdash \varphi : A \qquad \alpha \text{ Pos } \varphi}{\Sigma \vdash \nu\alpha\varphi : A}\ (\alpha \text{ guarded in } \varphi)$$

**Fig. 5.** Formation Rules of Formulae (where $A$, $B$ are pure types).

*Example 4.2.* (a) The formula $[\mathsf{hd}][\mathsf{a}] \Rightarrow \bigcirc[\mathsf{hd}][\mathsf{b}]$ means that if the head of a stream is $\mathsf{a}$, then its second element (the head of its tail) should be $\mathsf{b}$.

(b) On colists, we let $[\mathsf{hd}]\varphi := [\mathsf{fold}][\mathsf{in}_1][\pi_0]\varphi$ and $\bigcirc\psi := [\mathsf{fold}][\mathsf{in}_1][\pi_1][\mathsf{next}]\psi$.

(c) On (guarded) infinite binary trees over $A$, we also have a modality $[\mathsf{lbl}]\varphi := [\mathsf{fold}][\pi_0]\varphi : \mathsf{Tree}^{\mathsf{g}} A$ (provided $\varphi : A$). Moreover, we have modalities $\bigcirc_\ell$ and $\bigcirc_r$ defined on formulae $\varphi : \mathsf{Tree}^{\mathsf{g}} A$ as $\bigcirc_\ell\varphi := [\mathsf{fold}][\pi_1][\pi_0][\mathsf{next}]\varphi$ and $\bigcirc_r\varphi := [\mathsf{fold}][\pi_1][\pi_1][\mathsf{next}]\varphi$. Intuitively, $[\mathsf{lbl}]\varphi$ should hold on a tree $t$ over $A$ iff the root label of $t$ satisfies $\varphi$, and $\bigcirc_\ell\varphi$ (resp. $\bigcirc_r\varphi$) should hold on $t$ iff $\varphi$ holds on the left (resp. right) immediate subtree of $t$.

Formulae have fixpoints $\nu\alpha\varphi$. The rules of Fig. 5 thus allow for the formation of formulae with free typed propositional variables (ranged over by $\alpha, \beta, \dots$), and involve contexts $\Sigma$ of the form $\alpha_1 : A_1, \dots, \alpha_n : A_n$. In the formation of a fixpoint, the side condition "$\alpha$ guarded in $\varphi$" asks that each occurrence of $\alpha$ is beneath a $[\mathsf{next}]$ modality. Because we are ultimately interested in the *external* set-theoretic semantics of formulae, we assume a usual positivity condition of $\alpha$ in $\varphi$. It is defined with relations $\alpha$ Pos $\varphi$ and $\alpha$ Neg $\varphi$ (see [28, §B]). We just mention here that $[\mathsf{ev}(-)](-)$ is contravariant in its first argument. Note that $[\mathsf{box}]\varphi$ can only be formed for *closed* $\varphi$.

*Example 4.3.* (a) The modality $\Box$ makes it possible to express a range of safety properties. For instance, assuming $\varphi, \psi : \mathsf{Str}^{\mathsf{g}} A$, the formula $\Box(\psi \Rightarrow \bigcirc\varphi)$ is intended to hold on a stream $s = (s_i \mid i \geq 0)$ iff, for all $n \in \mathbb{N}$, if $(s_i \mid i \geq n)$ satisfies $\psi$, then $(s_i \mid i \geq n+1)$ satisfies $\varphi$.

(b) The modality $\Box$ has its two $\mathsf{CTL}$-like variants on $\mathsf{Tree}^{\mathsf{g}} A$, namely $\forall\Box\varphi := \nu\alpha.\ \varphi \wedge (\bigcirc_\ell\alpha \wedge \bigcirc_r\alpha)$ and $\exists\Box\varphi := \nu\alpha.\ \varphi \wedge (\bigcirc_\ell\alpha \vee \bigcirc_r\alpha)$. Assuming $\psi : A$, $\forall\Box[\mathsf{lbl}]\psi$ is intended to hold on a tree $t : \mathsf{Tree}^{\mathsf{g}} A$ iff all node-labels of $t$ satisfy $\psi$, while $\exists\Box[\mathsf{lbl}]\psi$ holds on $t$ iff $\psi$ holds on all nodes of *some* infinite path from the root of $t$.

| Name | Formulation | $[\pi_i]$ | [fold] | [next] | $[\mathsf{in}_i]$ | $[\mathsf{ev}(\psi)]$ | [box] | [hd] | $\bigcirc$ |
|---|---|---|---|---|---|---|---|---|---|
| (RM) | $\dfrac{\vdash \psi \Rightarrow \varphi}{\vdash [\triangle]\psi \Rightarrow [\triangle]\varphi}$ | ✓ | ✓ | ✓ | ✓ | ✓ | ✓ | ✓ | ✓ |
| (C) | $[\triangle]\varphi \wedge [\triangle]\psi \implies [\triangle](\varphi \wedge \psi)$ | ✓ | ✓ | ✓ | ✓ | ✓ | ✓ | ✓ | ✓ |
| (N) | $[\triangle]\top$ | ✓ | ✓ | ✓ | | ✓ | ✓ | ✓ | ✓ |
| (P) | $[\triangle]\bot \implies \bot$ | ✓ | ✓ | **(C)** | ✓ | | ✓ | ✓ | **(C)** |
| $(\mathsf{C}_\vee)$ | $[\triangle](\varphi \vee \psi) \implies [\triangle]\varphi \vee [\triangle]\psi$ | ✓ | ✓ | ✓ | ✓ | | ✓ | ✓ | ✓ |
| $(\mathsf{C}_\Rightarrow)$ | $([\triangle]\psi \Rightarrow [\triangle]\varphi) \Rightarrow [\triangle](\psi \Rightarrow \varphi)$ | ✓ | ✓ | **(C)** | | | ✓ | ✓ | **(C)** |

**Table 2.** Modal Axioms and Rules. Types are omitted in $\vdash$ and **(C)** marks axioms assumed for $\vdash_c$ but not for $\vdash$. Properties of the non-atomic [hd] and $\bigcirc$ are derived.

**Modal Theories.** Formulae are equipped with a modal deduction system which enters the type system via a subtyping relation (§5). For each pure type $A$, we have an intuitionistic theory $\vdash^A$ (the general case) and a classical theory $\vdash^A_c$ (which is only assumed under $\blacksquare$/[box]), summarized in Fig. 6 and Table 2 (where we also give properties of the derived modalities [hd], $\bigcirc$). In any case, $\vdash^A_{(c)} \varphi$ is only defined when $\vdash \varphi : A$ (and so when $\varphi$ has no free propositional variable).

Fixpoints $\nu\alpha\varphi$ are equipped with their usual Kozen axioms [43]. The atomic modalities $[\pi_i]$, [fold], [next], $[\mathsf{in}_i]$ and [box] have deterministic branching (see Fig. 12, §7). We can get the axioms of the intuitionistic (normal) modal logic **IK** [56] (see also e.g. [60,48]) for $[\pi_i]$, [fold] and [box] but not for $[\mathsf{in}_i]$ nor for the intuitionistic [next]. For [next], in the intuitionistic case this is due to semantic issues with step indexing (discussed in §7) which are absent from the classical case. As for $[\mathsf{in}_i]$, we have a logical theory allowing for a coding of finite base types as finite sum types, which allows to derive, for a finite base type B:

$$\vdash^{\mathsf{B}} \quad \bigvee_{\mathsf{a}\in\mathsf{B}} \Big([\mathsf{a}] \quad \wedge \quad \bigwedge_{\substack{\mathsf{b}\in\mathsf{B}\\ \mathsf{b}\neq\mathsf{a}}} \neg[\mathsf{b}]\Big)$$

**Definition 4.4 (Modal Theories).** *For each pure type $A$, the* intuitionistic *and* classical *modal theories $\vdash^A \varphi$ and $\vdash^A_c \varphi$ (where $\vdash \varphi : A$) are defined by mutual induction:*

– *The theory $\vdash^A$ is deduction for intuitionistic propositional logic augmented with the check-marked (✓) axioms and rules of Table 2 and the axioms and rules of Fig. 6 (for $\vdash^A$).*
– *The theory $\vdash^A_c$ is $\vdash^A$ augmented with the axioms* (P) *and* $(\mathsf{C}_\Rightarrow)$ *for* [next] *and with the axiom* (CL) *(Fig. 6).*

For example, we have $\vdash^{\mathsf{Str}^{\mathsf{g}}\,A} \Box\psi \Rightarrow (\psi \wedge \bigcirc\Box\psi)$ and $\vdash^{\mathsf{Str}^{\mathsf{g}}\,A} (\psi \wedge \bigcirc\Box\psi) \Rightarrow \Box\psi$.

$$\frac{\vdash^{B} \psi \Rightarrow \phi \qquad \vdash \varphi : A}{\vdash^{B \to A} [\mathsf{ev}(\phi)]\varphi \Rightarrow [\mathsf{ev}(\psi)]\varphi} \qquad \frac{}{\vdash^{B \to A} ([\mathsf{ev}(\psi_0)]\varphi \wedge [\mathsf{ev}(\psi_1)]\varphi) \Rightarrow [\mathsf{ev}(\psi_0 \vee \psi_1)]\varphi}$$

$$\frac{}{\vdash_c^{A} ((\varphi \Rightarrow \psi) \Rightarrow \varphi) \Rightarrow \varphi}\,(\mathsf{CL}) \qquad \frac{\vdash_c^{A} \varphi}{\vdash^{\blacksquare A} [\mathsf{box}]\varphi} \qquad \frac{}{\vdash^{A_0 + A_1} \big([\mathsf{in}_0]\top \vee [\mathsf{in}_1]\top\big) \wedge \neg\big([\mathsf{in}_0]\top \wedge [\mathsf{in}_1]\top\big)}$$

$$\frac{}{\vdash^{A_0 + A_1} ([\mathsf{in}_i]\top) \Rightarrow (\neg[\mathsf{in}_i]\varphi \Leftrightarrow [\mathsf{in}_i]\neg\varphi)} \qquad \frac{}{\vdash^{A} \nu\alpha\varphi \Rightarrow \varphi[\nu\alpha\varphi/\alpha]} \qquad \frac{\vdash^{A} \psi \Rightarrow \varphi[\psi/\alpha]}{\vdash^{A} \psi \Rightarrow \nu\alpha\varphi}$$

**Fig. 6.** Modal Axioms and Rules.

$$\frac{}{T \le |T|} \qquad \frac{}{A \le \{A \mid \top\}} \qquad \frac{\vdash^{A} \varphi \Rightarrow \psi}{\{A \mid \varphi\} \le \{A \mid \psi\}} \qquad \frac{\vdash_c^{A} \varphi \Rightarrow \psi}{\{\blacksquare A \mid [\mathsf{box}]\varphi\} \le \{\blacksquare A \mid [\mathsf{box}]\psi\}}$$

$$\frac{}{\{\blacktriangleright A \mid [\mathsf{next}]\varphi\} \equiv \blacktriangleright \{A \mid \varphi\}} \qquad \frac{}{\{B \to A \mid [\mathsf{ev}(\psi)]\varphi\} \equiv \{B \mid \psi\} \to \{A \mid \varphi\}}$$

**Fig. 7.** Subtyping Rules (excerpt).

### 5 A Temporally Refined Type System

*Temporal refinement types* (or *types*), notation $T, U, V$, etc., are defined by:

$$T, U ::= A \mid \{A \mid \varphi\} \mid T + T \mid T \times T \mid T \to T \mid \blacktriangleright T \mid \blacksquare T$$

where $\vdash \varphi : A$ and, in the case of $\blacksquare T$, the type $T$ has no free type variable. So types are built from (closed) pure types $A$ and temporal refinements $\{A \mid \varphi\}$. They allow for all the type constructors of pure types.

As a refinement type $\{A \mid \varphi\}$ intuitively represents a subset of the inhabitants of $A$, it is natural to equip our system with a notion of *subtyping*. In addition to the usual rules for product, arrow and sum types, our subtyping relation is made of two more ingredients. The first follows the principle that our refinement type system is meant to prove properties of programs, and not to type more programs, so that (say) a type of the form $\{A \mid \varphi\} \to \{B \mid \psi\}$ is a subtype of $A \to B$. We formalize this with the notion of *underlying pure type* $|T|$ of a type $T$. The second ingredient is the modal theory $\vdash^{A} \varphi$ of §4. The subtyping rules concerning refinements are given in Fig. 7, where $T \equiv U$ enforces both $T \le U$ and $U \le T$. The full set of rules is given in [28, §C]. Notice that subtyping does not incorporate (un)folding of guarded recursive types.

*Typing* for refinement types is given by the rules of Fig. 8, together with the rules of §3 *extended to refinement types*, where $T$ is *constant* if $|T|$ is constant. Modalities $[\pi_i]$, $[\mathsf{in}_i]$, $[\mathsf{fold}]$ and $[\mathsf{ev}(-)]$ (but not $[\mathsf{next}]$) have introduction rules extending those of the corresponding term formers.

$$(\textsc{Pi}_i\text{-I})\ \frac{\mathcal{E} \vdash M_i : \{A_i \mid \varphi\} \qquad \mathcal{E} \vdash M_{1-i} : A_{1-i}}{\mathcal{E} \vdash \langle M_0, M_1\rangle : \{A_0 \times A_1 \mid [\pi_i]\varphi\}} \qquad (\textsc{Pi}_i\text{-E})\ \frac{\mathcal{E} \vdash M : \{A_0 \times A_1 \mid [\pi_i]\varphi\}}{\mathcal{E} \vdash \pi_i(M) : \{A_i \mid \varphi\}}$$

$$(\textsc{Ev-I})\ \frac{\mathcal{E}, x : \{B \mid \psi\} \vdash M : \{A \mid \varphi\}}{\mathcal{E} \vdash \lambda x.M : \{B \to A \mid [\mathsf{ev}(\psi)]\varphi\}} \qquad (\textsc{Ev-E})\ \frac{\mathcal{E} \vdash M : \{B \to A \mid [\mathsf{ev}(\psi)]\varphi\} \qquad \mathcal{E} \vdash N : \{B \mid \psi\}}{\mathcal{E} \vdash MN : \{A \mid \varphi\}}$$

$$(\textsc{Fd-I})\ \frac{\mathcal{E} \vdash M : \{A[\mathsf{Fix}(X).A/X] \mid \varphi\}}{\mathcal{E} \vdash \mathsf{fold}(M) : \{\mathsf{Fix}(X).A \mid [\mathsf{fold}]\varphi\}} \qquad (\textsc{Fd-E})\ \frac{\mathcal{E} \vdash M : \{\mathsf{Fix}(X).A \mid [\mathsf{fold}]\varphi\}}{\mathcal{E} \vdash \mathsf{unfold}(M) : \{A[\mathsf{Fix}(X).A/X] \mid \varphi\}}$$

$$(\textsc{Inj}_i\text{-E})\ \frac{\mathcal{E} \vdash M : \{A_0 + A_1 \mid [\mathsf{in}_i]\varphi\} \qquad \mathcal{E}, x : \{A_i \mid \varphi\} \vdash N_i : U \qquad \mathcal{E}, x : A_{1-i} \vdash N_{1-i} : U}{\mathcal{E} \vdash \mathsf{case}\, M \,\mathsf{of}\, (x.N_0 | x.N_1) : U}$$

$$(\vee\text{-E})\ \frac{\mathcal{E} \vdash M : \{A \mid \varphi_0 \vee \varphi_1\} \qquad \overset{\text{for } i \in \{0,1\},}{\mathcal{E}, x : \{A \mid \varphi_i\} \vdash N : U}}{\mathcal{E} \vdash N[M/x] : U} \qquad (\textsc{Inj}_i\text{-I})\ \frac{\mathcal{E} \vdash M : \{A_i \mid \varphi\}}{\mathcal{E} \vdash \mathsf{in}_i(M) : \{A_0 + A_1 \mid [\mathsf{in}_i]\varphi\}}$$

$$(\textsc{MP})\ \frac{\mathcal{E} \vdash M : \{A \mid \psi \Rightarrow \varphi\} \quad \mathcal{E} \vdash M : \{A \mid \psi\}}{\mathcal{E} \vdash M : \{A \mid \varphi\}} \qquad (\textsc{ExF})\ \frac{\mathcal{E} \vdash M : \{A \mid \bot\} \quad \mathcal{E} \vdash N : |U|}{\mathcal{E} \vdash N : U}$$

$$(\textsc{Sub})\ \frac{\mathcal{E} \vdash M : T \quad T \leq U}{\mathcal{E} \vdash M : U}$$

**Fig. 8.** Typing Rules for Refined Modal Types.

*Example 5.1.* Since $\varphi \Rightarrow \psi \Rightarrow (\varphi \wedge \psi)$ and using two times the rule (MP), we get the first derived rule below, from which we can deduce the second one:

$$\frac{\mathcal{E} \vdash M : \{A \mid \varphi\} \quad \mathcal{E} \vdash M : \{A \mid \psi\}}{\mathcal{E} \vdash M : \{A \mid \varphi \wedge \psi\}} \qquad \frac{\mathcal{E} \vdash M : \{A \mid \varphi\} \quad \mathcal{E} \vdash N : \{B \mid \psi\}}{\mathcal{E} \vdash \langle M, N\rangle : \{A \times B \mid [\pi_0]\varphi \wedge [\pi_1]\psi\}}$$

*Example 5.2.* We have the following derived rules:

$$\frac{\mathcal{E} \vdash M : \{\mathsf{Str}^{\mathsf{g}}\, A \mid \Box\varphi\}}{\mathcal{E} \vdash M : \{\mathsf{Str}^{\mathsf{g}}\, A \mid \varphi \wedge \bigcirc\Box\varphi\}} \quad \text{and} \quad \frac{\mathcal{E} \vdash M : \{\mathsf{Str}^{\mathsf{g}}\, A \mid \varphi \wedge \bigcirc\Box\varphi\}}{\mathcal{E} \vdash M : \{\mathsf{Str}^{\mathsf{g}}\, A \mid \Box\varphi\}}$$

*Example 5.3.* We have $\mathsf{Cons}^{\mathsf{g}} : A \to \blacktriangleright \{\mathsf{Str}^{\mathsf{g}}\, A \mid \varphi\} \to \{\mathsf{Str}^{\mathsf{g}}\, A \mid \bigcirc\varphi\}$ as well as $\mathsf{tl}^{\mathsf{g}} : \{\mathsf{Str}^{\mathsf{g}}\, A \mid \bigcirc\varphi\} \to \blacktriangleright \{\mathsf{Str}^{\mathsf{g}}\, A \mid \varphi\}$.

*Example 5.4 ("Always" (□) on Guarded Streams).* The refined types of $\mathsf{Cons}^{\mathsf{g}}$, $\mathsf{hd}^{\mathsf{g}}$, $\mathsf{tl}^{\mathsf{g}}$ and $\mathsf{map}^{\mathsf{g}}$ mentioned in §2 are easy to derive. We also have the type

$$\{\mathsf{Str}^{\mathsf{g}}\, A \mid \Box[\mathsf{hd}]\varphi_0\} \longrightarrow \{\mathsf{Str}^{\mathsf{g}}\, A \mid \Box[\mathsf{hd}]\varphi_1\} \longrightarrow \{\mathsf{Str}^{\mathsf{g}}\, A \mid \Box([\mathsf{hd}]\varphi_0 \vee [\mathsf{hd}]\varphi_1)\}$$

for the $\mathsf{merge}^{\mathsf{g}}$ function which takes two guarded streams and interleaves them:

$$\begin{array}{ll} \mathsf{merge}^{\mathsf{g}} : & \mathsf{Str}^{\mathsf{g}}\, A \longrightarrow \mathsf{Str}^{\mathsf{g}}\, A \longrightarrow \mathsf{Str}^{\mathsf{g}}\, A \\ & := \mathsf{fix}(g).\lambda s_0.\lambda s_1.\ (\mathsf{hd}^{\mathsf{g}}\ s_0) ::^{\mathsf{g}} \mathsf{next}\big((\mathsf{hd}^{\mathsf{g}}\ s_1) ::^{\mathsf{g}} (g \circledast (\mathsf{tl}^{\mathsf{g}}\ s_0) \circledast (\mathsf{tl}^{\mathsf{g}}\ s_1))\big) \end{array}$$

## 6 The Full System

The system presented so far has only one form of fixpoints in formulae ($\nu\alpha\varphi$). We now present our full system, which also handles least fixpoints ($\mu\alpha\varphi$) and thus liveness properties. A key role is played by *polynomial* guarded recursive types, that we discuss first.

$$(\mu\text{-F})\ \frac{\Sigma, \alpha : A \vdash \varphi : A}{\Sigma \vdash \mu\alpha\varphi : A} \qquad \frac{\Sigma, \alpha : A \vdash \varphi : A}{\Sigma \vdash \mu^{\mathtt{t}}\alpha\varphi : A} \qquad \frac{\Sigma, \alpha : A \vdash \varphi : A}{\Sigma \vdash \nu^{\mathtt{t}}\alpha\varphi : A}$$

**Fig. 9.** Extended Formation Rules of Formulae (with $\alpha$ Pos $\varphi$ and $\alpha$ guarded in $\varphi$).

$$\frac{}{\vdash^A \varphi[\mu\alpha\varphi/\alpha] \Rightarrow \mu\alpha\varphi} \qquad \frac{\vdash^A \varphi[\psi/\alpha] \Rightarrow \psi}{\vdash^A \mu\alpha\varphi \Rightarrow \psi}$$

$$\frac{}{\vdash^A \theta^{\mathtt{t}+1}\alpha\varphi \Leftrightarrow \varphi[\theta^{\mathtt{t}}\alpha\varphi/\alpha]} \qquad \frac{}{\vdash^A \mu^{0}\alpha\varphi \Leftrightarrow \bot} \qquad \frac{}{\vdash^A \nu^{0}\alpha\varphi \Leftrightarrow \top}$$

$$\frac{[\![\mathtt{t}]\!] \leq [\![\mathtt{u}]\!]}{\vdash^A \mu^{\mathtt{t}}\alpha\varphi \Rightarrow \mu^{\mathtt{u}}\alpha\varphi} \qquad \frac{}{\vdash^A \mu^{\mathtt{t}}\alpha\varphi \Rightarrow \mu\alpha\varphi} \qquad \frac{[\![\mathtt{t}]\!] \geq [\![\mathtt{u}]\!]}{\vdash^A \nu^{\mathtt{t}}\alpha\varphi \Rightarrow \nu^{\mathtt{u}}\alpha\varphi} \qquad \frac{}{\vdash^A \nu\alpha\varphi \Rightarrow \nu^{\mathtt{t}}\alpha\varphi}$$

**Fig. 10.** Extended Modal Axioms and Rules (with $A$ a pure type and $\theta$ either $\mu$ or $\nu$).

**Strictly Positive and Polynomial Types.** *Strictly positive types* (notation $P^+, Q^+$, etc.) are given by

$$P^+ ::= A \mid X \mid \blacktriangleright P^+ \mid P^+ + P^+ \mid P^+ \times P^+ \mid \mathsf{Fix}(X).P^+ \mid B \to P^+$$

where $A$, $B$ are (closed) *constant* pure types. Strictly positive types are a convenient generalization of polynomial types. A guarded recursive type $\mathsf{Fix}(X).P(X)$ is *polynomial* if $P(X)$ is induced by

$$P(X) ::= A \ \mid\ \blacktriangleright X \ \mid\ P(X) + P(X) \ \mid\ P(X) \times P(X) \ \mid\ B \to P(X)$$

where $A$, $B$ are (closed) *constant* pure types. Note that if $\mathsf{Fix}(X).P(X)$ is polynomial, $X$ cannot occur on the left of an arrow ($\to$) in $P(X)$. We say that $\mathsf{Fix}(X).P(X)$ (resp. $P^+$) is *finitary* polynomial (resp. *finitary* strictly positive) if $B$ is a finite base type (see Ex. 3.1) in the above grammars. The set-theoretic counterpart of our polynomial recursive types are the *exponent* polynomial functors of [31], which all have final **Set**-coalgebras (see e.g. [31, Cor. 4.6.3]).

*Example 6.1.* For $A$ a constant pure type, e.g. $\mathsf{Str}^{\mathsf{g}} A$, $\mathsf{CoList}^{\mathsf{g}} A$ and $\mathsf{Tree}^{\mathsf{g}} A$ as well as $\mathsf{Str}^{\mathsf{g}}(\mathsf{Str}\, A)$, $\mathsf{CoList}^{\mathsf{g}}(\mathsf{Str}\, A)$ and $\mathsf{Res}^{\mathsf{g}} A$ (with $\mathtt{I}$, $\mathtt{O}$ constant) are polynomial. More generally, polynomial types include all recursive types $\mathsf{Fix}(X).P(X)$ where $P(X)$ is of the form $\sum_{i=0}^{n} A_i \times (\blacktriangleright X)^{B_i}$ with $A_i$, $B_i$ constant. The non-strictly positive recursive type $\mathsf{Rou}^{\mathsf{g}} A$ of Ex. 3.2, used in Hofmann's breadth-first traversal (see e.g. [10]), is *not* polynomial.

**The Full Temporal Modal Logic.** We assume given a first-order signature of *iteration terms* (notation $\mathtt{t}, \mathtt{u}$, etc.), with *iteration variables* $k, \ell$, etc., and for each iteration term $\mathtt{t}(k_1, \ldots, k_m)$ with variables as shown, a given primitive recursive function $[\![\mathtt{t}]\!] : \mathbb{N}^m \to \mathbb{N}$. We assume a term $\mathtt{0}$ for $0 \in \mathbb{N}$ and a term $k\mathtt{+1}$ for the successor function $n \in \mathbb{N} \mapsto n + 1 \in \mathbb{N}$.

The formulae of the *full temporal modal logic* extend those of Fig. 5 with least fixpoints $\mu\alpha\varphi$ and with *approximated fixpoints* $\mu^{\mathtt{t}}\alpha\varphi$ and $\nu^{\mathtt{t}}\alpha\varphi$ where $\mathtt{t}$ is an iteration term. The additional formation rule for formulae are given in Fig. 9. We use $\theta$ as a generic notation for $\mu$ and $\nu$. Least fixpoints $\mu\alpha\varphi$ are equipped with their usual Kozen axioms. In addition, iteration formulae $\nu^{\mathtt{t}}\alpha\varphi(\alpha)$ and $\mu^{\mathtt{t}}\alpha\varphi(\alpha)$ have axioms expressing that they are indeed iterations of $\varphi(\alpha)$ from resp. $\top$ and $\bot$. A fixpoint logic with iteration variables was already considered in [63].

**Definition 6.2 (Full Modal Theories).** *The* full *intuitionistic and classical modal theories (still denoted* $\vdash^{A}$ *and* $\vdash^{A}_{\mathrm{c}}$*) are defined by extending Def. 4.4 with the axioms and rules of Fig. 10.*

*Example 6.3.* Least fixpoints allow us to define liveness properties. On streams and colists, we have $\Diamond\varphi := \mu\alpha.\ \varphi \vee \bigcirc\alpha$ and $\varphi \ \mathsf{U}\ \psi := \mu\alpha.\ \psi \vee (\varphi \wedge \bigcirc\alpha)$. On trees, we have the $\mathsf{CTL}$-like $\exists\Diamond\varphi := \mu\alpha.\ \varphi \vee (\bigcirc_{\ell}\alpha \vee \bigcirc_{r}\alpha)$ and $\forall\Diamond\varphi := \mu\alpha.\ \varphi \vee (\bigcirc_{\ell}\alpha \wedge \bigcirc_{r}\alpha)$. The formula $\exists\Diamond\varphi$ is intended to hold on a tree if there is a finite path which leads to a subtree satisfying $\varphi$, while $\forall\Diamond\varphi$ is intended to hold if every infinite path crosses a subtree satisfying $\varphi$.

*Remark 6.4.* On *finitary trees* (as in Ex. 6.1 but with $A_i$, $B_i$ finite base types), we have all formulae of the modal $\mu$-calculus. For this fragment, satisfiability is decidable (see e.g. [16]), as well as the *classical* theory $\vdash_{\mathrm{c}}$ by completeness of Kozen's axiomatization [68] (see [58] for completeness results on fragments of the $\mu$-calculus).

**The Safe and Smooth Fragments.** We now discuss two related but distinct fragments of the temporal modal logic. Both fragments directly impact the refinement type system by allowing for more typing rules.

The *safe* fragment plays a crucial role, because it reconciles the internal and external semantics of our system (see §7). It gives subtyping rules for $\blacksquare$ (Fig. 11), which makes available the comonad structure of $\blacksquare$ on $[\mathsf{box}]\varphi$ when $\varphi$ is safe.

**Definition 6.5 (Safe Formula).** *Say* $\alpha_1 : A_1, \ldots, \alpha_n : A_n \vdash \varphi : A$ *is* safe *if*

*(i) the types* $A_1, \ldots, A_n, A$ *are strictly positive, and*
*(ii) for each occurrence in* $\varphi$ *of a modality* $[\mathsf{ev}(\psi)]$*, the formula* $\psi$ *is closed, and*
*(iii) each occurrence in* $\varphi$ *of a least fixpoint (*$\mu\alpha(-)$*) and of an implication (*$\Rightarrow$*) is guarded by a* $[\mathsf{box}]$*.*

Note that the safe restriction imposes no condition on approximated fixpoints $\theta^{\mathtt{t}}\alpha$. Recalling that the theory under a $[\mathsf{box}]$ is $\vdash^{A}_{\mathrm{c}}$, the only propositional connectives accessible to $\vdash^{A}$ in safe formulae are those on which $\vdash^{A}$ and $\vdash^{A}_{\mathrm{c}}$ coincide. The formula $[\neg\mathsf{nil}] = [\mathsf{fold}][\mathsf{in}_1]\top$ is safe. Moreover:

*Example 6.6.* Any formula without fixpoint nor $[\mathsf{ev}(-)]$ is equivalent in $\vdash_{\mathrm{c}}$ to a safe one. It $\varphi$ is safe, then so are $[\mathsf{hd}]\varphi$, $[\mathsf{lbl}]\varphi$, as well as $\triangle\varphi$ (for $\triangle \in \{\Box, \forall\Box, \exists\Box\}$) and $[\mathsf{box}]\triangle\varphi$ (for $\triangle \in \{\Diamond, \exists\Diamond, \forall\Diamond\}$).

**Definition 6.7 (Smooth Formula).** *A formula* $\alpha_1 : A_1, \ldots, \alpha_n : A_n \vdash \varphi : A$ *is* smooth *if*

*(i)* *the types* $A_1, \ldots, A_n, A$ *are* finitary *strictly positive, and*
*(ii)* *for each occurrence in* $\varphi$ *of a modality* $[\mathsf{ev}(\psi)]$*, the formula* $\psi$ *is closed, and*
*(iii)* $\varphi$ *is* alternation-free*: for* $\theta, \theta' \in \{\mu, \nu\}$*, (1) if* $\theta\beta_0\psi_0$ *is a subformula of* $\varphi$*, and* $\theta'\beta_1\psi_1$ *is a subformula of* $\psi_0$ *s.t.* $\beta_0$ *occurs free in* $\psi_1$*, then* $\theta = \theta'$*, (2) if some* $\alpha_i$ *occurs in two subformulae* $\theta\beta_0\psi_0$ *and* $\theta'\beta_1\psi_1$ *of* $\varphi$*, then* $\theta = \theta'$*, and (3) if some* $\alpha_i$ *occurs in a subformula* $\theta'\beta\psi$ *of* $\varphi$*, then* $\alpha_i$ Pos $\psi$*.*

Our notion of alternation freedom is adapted from [16], in which propositional (fixpoint) variables are always positive. Note that the smooth restriction imposes no further conditions on approximated fixpoints $\theta^{\mathsf{t}}\alpha$. In the smooth fragment, greatest and least fixpoints can be thought about resp. as

$$\bigwedge_{m \in \mathbb{N}} \varphi^m(\top) \qquad \text{and} \qquad \bigvee_{m \in \mathbb{N}} \varphi^m(\bot)$$

Iteration terms allow for formal reasoning about such unfoldings. Assuming $[\![\mathsf{t}]\!] = m \in \mathbb{N}$, the formula $\nu^{\mathsf{t}}\alpha\varphi(\alpha)$ (resp. $\mu^{\mathsf{t}}\alpha\varphi(\alpha)$) can be read as $\varphi^m(\top)$ (resp. $\varphi^m(\bot)$). This gives the rules ($\nu$-I) and ($\mu$-E) (Fig. 11), which allow for reductions to the safe case (see examples in §8).

*Remark 6.8.* It is well-known (see e.g. [16, §4.1]) that on *finitary trees* (see Rem. 6.4) the alternation-free fragment is equivalent to *Weak* MSO (MSO with second-order variables restricted to *finite* sets). In the case of streams $\mathtt{Str}\,\mathtt{B}$ (for a finite base type $\mathtt{B}$), Weak MSO is in turn equivalent to the full modal $\mu$-calculus. In particular, the alternation-free fragment contains all the *flat* fixpoints of [58] and thus LTL on $\mathtt{Str}\,\mathtt{B}$ and CTL on $\mathtt{Tree}\,\mathtt{B}$ and on $\mathtt{Res}\,\mathtt{B}$ with $\mathtt{I}$, $\mathtt{O}$, $\mathtt{B}$ finite base types. A typical property on $\mathtt{Tree}\,\mathtt{B}$ which *cannot* be expressed with alternation-free formulae is "there is an infinite path with infinitely many occurrences of $\mathtt{b}$" for a fixed $\mathtt{b} : \mathtt{B}$ (see e.g. [16, §2.2]).

*Example 6.9.* Any formula without fixpoint nor $[\mathsf{ev}(-)]$ is smooth. It $\varphi$ is smooth, then so are $[\mathsf{hd}]\varphi$, $[\mathsf{lbl}]\varphi$ and $\triangle\varphi$ for $\triangle \in \{\Box, \forall\Box, \exists\Box, \Diamond, \exists\Diamond, \forall\Diamond\}$.

**The Full System.** We extend the types of §5 with universal quantification over iteration variables ($\forall k \cdot T$). The type system of §5 is extended with the rules of Fig. 11.

*Example 6.10.* The logical rules of Fig. 10 give the following derived typing rules (where $\beta$ Pos $\gamma$):

$$(\mu\text{-I})\ \frac{\mathcal{E} \vdash M : \{\blacksquare A \mid [\mathsf{box}]\gamma[\mu^{\mathsf{t}}\alpha\varphi/\beta]\}}{\mathcal{E} \vdash M : \{\blacksquare A \mid [\mathsf{box}]\gamma[\mu\alpha\varphi/\beta]\}} \qquad (\nu\text{-E})\ \frac{\mathcal{E} \vdash M : \{\blacksquare A \mid [\mathsf{box}]\gamma[\nu\alpha\varphi/\beta]\}}{\mathcal{E} \vdash M : \{\blacksquare A \mid [\mathsf{box}]\gamma[\nu^{\mathsf{t}}\alpha\varphi/\beta]\}}$$

$$\frac{\varphi \text{ safe}}{\{\blacksquare A \mid [\mathsf{box}]\varphi\} \equiv \blacksquare\{A \mid \varphi\}} \qquad \frac{}{\forall k \cdot \blacktriangleright T \equiv \blacktriangleright \forall k \cdot T}$$

$$(\forall\text{-I})\ \frac{\mathcal{E} \vdash M : T}{\mathcal{E} \vdash M : \forall k \cdot T} \qquad (\forall\text{-CI})\ \frac{\mathcal{E} \vdash M : T[\mathsf{0}/k] \qquad \mathcal{E} \vdash M : T[k{+}\mathsf{1}/k]}{\mathcal{E} \vdash M : \forall k \cdot T}$$

$$(\nu\text{-I})\ \frac{\mathcal{E} \vdash M : \left\{\blacksquare A \;\middle|\; [\mathsf{box}]\gamma[\nu^{\ell}\alpha\psi/\beta]\right\}}{\mathcal{E} \vdash M : \{\blacksquare A \mid [\mathsf{box}]\gamma[\nu\alpha\psi/\beta]\}} \qquad (\forall\text{-E})\ \frac{\mathcal{E} \vdash M : \forall k \cdot T}{\mathcal{E} \vdash M : T[\mathsf{t}/k]}$$

$$(\mu\text{-E})\ \frac{\mathcal{E} \vdash M : \{\blacksquare A \mid [\mathsf{box}]\gamma[\mu\alpha\psi/\beta]\} \qquad \mathcal{E}, x : \left\{\blacksquare A \;\middle|\; [\mathsf{box}]\gamma[\mu^{\ell}\alpha\psi/\beta]\right\} \vdash N : U}{\mathcal{E} \vdash N[M/x] : U}$$

**Fig. 11.** Extended (Sub)Typing Rules for Refinement Types (where $k$ is not free in $\mathcal{E}$ in ($\forall$-I) & ($\forall$-CI), $\ell$ is fresh in ($\nu$-I) & ($\mu$-E), $\theta\alpha\psi$ and $\gamma$ are smooth, and $\beta$ Pos $\gamma$).

### 7 Semantics

We present the main ingredients of the semantics of our type system. We take as base the denotational semantics of guarded recursion in the topos of trees.

**Denotational Semantics in the Topos of Trees.** The *topos of trees* $\mathcal{S}$ provides a natural model of guarded recursion [13]. Formally, $\mathcal{S}$ is the category of presheaves over $(\mathbb{N} \setminus \{0\}, \leq)$. In words, the objects of $\mathcal{S}$ are indexed sets $X = (X(n))_{n>0}$ equipped with *restriction maps* $r^X_n : X(n+1) \to X(n)$. Excluding 0 from the indexes is a customary notational convenience ([13]). The morphisms from $X$ to $Y$ are families of functions $f = (f_n : X(n) \to Y(n))_{n>0}$ which commute with restriction, that is $f_n \circ r^X_n = r^Y_n \circ f_{n+1}$. As any presheaf category, $\mathcal{S}$ has (pointwise) limits and colimits, and is Cartesian closed (see e.g. [47, §I.6]). We write $\boldsymbol{\Gamma} : \mathcal{S} \to \mathbf{Set}$ for the *global section functor*, which takes $X$ to $\mathcal{S}[\mathbf{1}, X]$, the set of morphisms $\mathbf{1} \to X$ in $\mathcal{S}$, where $\mathbf{1} = (\{\bullet\})_{n>0}$ is terminal in $\mathcal{S}$.

A typed term $\mathcal{E} \vdash M : T$ is to be interpreted in $\mathcal{S}$ as a morphism

$$[\![M]\!] : [\![|\mathcal{E}|]\!] \longrightarrow [\![|T|]\!]$$

where $[\![|\mathcal{E}|]\!] = [\![|T_1|]\!] \times \cdots \times [\![|T_n|]\!]$ for $\mathcal{E} = x_1 : T_1, \ldots, x_n : T_n$. In particular, a closed term $M : T$ is to be interpreted as a global section $[\![M]\!] \in \boldsymbol{\Gamma}[\![|T|]\!]$. The $\times/+/\to$ fragment of the calculus is interpreted by the corresponding structure in $\mathcal{S}$. The $\blacktriangleright$ modality is interpreted by the functor $\blacktriangleright : \mathcal{S} \to \mathcal{S}$ of [13]. This functor shifts indexes by 1 and inserts a singleton set $\mathbf{1}$ at index 1. The term constructor $\mathsf{next}$ is interpreted by the natural map with component $\mathsf{next}^X : X \to \blacktriangleright X$ as in

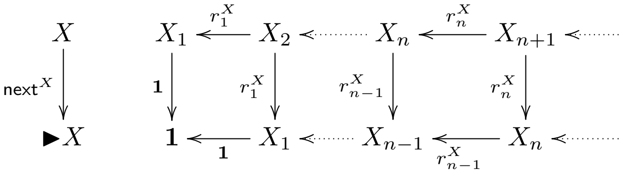

$$\{\!|[\pi_i]\varphi|\!\} := \{x \in \boldsymbol{\Gamma}[\![A_0 \times A_1]\!] \mid \pi_i \circ x \in \{\!|\varphi|\!\}\} \qquad \{\!|[\mathsf{next}]\varphi|\!\} := \{\mathsf{next} \circ x \in \boldsymbol{\Gamma}[\![\blacktriangleright A]\!] \mid x \in \{\!|\varphi|\!\}\}$$
$$\{\!|[\mathsf{fold}]\varphi|\!\} := \{x \in \boldsymbol{\Gamma}[\![\mathsf{Fix}(X).A]\!] \mid \mathsf{unfold} \circ x \in \{\!|\varphi|\!\}\} \quad \{\!|[\mathsf{box}]\varphi|\!\} := \{x \in \boldsymbol{\Gamma}[\![\blacksquare A]\!] \mid x_1(\bullet) \in \{\!|\varphi|\!\}\}$$
$$\{\!|[\mathsf{in}_i]\varphi|\!\} := \{x \in \boldsymbol{\Gamma}[\![A_0 + A_1]\!] \mid \exists y \in \boldsymbol{\Gamma}[\![A_i]\!](x = \mathsf{in}_i \circ y \;\text{ and }\; y \in \{\!|\varphi|\!\})\}$$
$$\{\!|[\mathsf{ev}(\psi)]\varphi|\!\} := \{x \in \boldsymbol{\Gamma}[\![B \to A]\!] \mid \forall y \in \boldsymbol{\Gamma}[\![B]\!](y \in \{\!|\psi|\!\} \implies \mathsf{ev} \circ \langle x, y\rangle \in \{\!|\varphi|\!\})\}$$

**Fig. 12.** External Semantics (for closed formulae).

The guarded fixpoint combinator fix is interpreted by the morphism $\mathsf{fix}^X : X^{\blacktriangleright X} \to X$ of [13, Thm. 2.4].

The constant type modality $\blacksquare$ is interpreted as the comonad $\boldsymbol{\Delta\Gamma} : \mathcal{S} \to \mathcal{S}$, where the left adjoint $\boldsymbol{\Delta} : \mathbf{Set} \to \mathcal{S}$ is the *constant object functor*, which takes a set $S$ to the constant family $(S)_{n>0}$. In words, all components $[\![\blacksquare A]\!](n)$ are equal to $\boldsymbol{\Gamma}[\![A]\!]$, and the restriction maps of $[\![\blacksquare A]\!]$ are identities. In particular, a global section $x \in \boldsymbol{\Gamma}[\![\blacksquare A]\!]$ is a constant family $(x_n)_n$ describing a unique global section $x_{n+1}(\bullet) = x_n(\bullet) \in \boldsymbol{\Gamma}[\![A]\!]$. We refer to [18] and [28, §D] for the interpretation of prev, box and unbox. Just note that the unit $\eta : \mathrm{Id}_{\mathbf{Set}} \to \boldsymbol{\Gamma\Delta}$ is an iso.

Together with an interpretation of guarded recursive types, this gives a denotational semantics of the pure calculus of §3. See [13,18] for details. We write $\mathsf{fold} : [\![A[\mathsf{Fix}(X).A/X]]\!] \to [\![\mathsf{Fix}(X).A]\!]$ and $\mathsf{unfold} : [\![\mathsf{Fix}(X).A]\!] \to [\![A[\mathsf{Fix}(X).A/X]]\!]$ for the two components of the iso $[\![\mathsf{Fix}(X).A]\!] \simeq [\![A[\mathsf{Fix}(X).A/X]]\!]$.

**External Semantics.** Møgelberg [50] has shown that for polynomial types such as $\mathsf{Str}^{\mathsf{g}}\, B$ with $B$ a constant type, the set of global sections $\boldsymbol{\Gamma}[\![\mathsf{Str}^{\mathsf{g}}\, B]\!]$ is equipped with the usual final coalgebra structure of streams over $B$ in **Set**. To each polynomial recursive type $\mathsf{Fix}(X).P(X)$, we associate a polynomial functor $P_{\mathbf{Set}} : \mathbf{Set} \to \mathbf{Set}$ in the obvious way.

**Theorem 7.1 ([50] (see also [18])).** *If* $\mathsf{Fix}(X).P(X)$ *is polynomial, then the set* $\boldsymbol{\Gamma}[\![\mathsf{Fix}(X).P(X)]\!]$ *carries a final* **Set***-coalgebra structure for* $P_{\mathbf{Set}}$*.*

We devise a **Set** interpretation $\{\!|\varphi|\!\} \in \mathcal{P}(\boldsymbol{\Gamma}[\![A]\!])$ of formulae $\varphi : A$. We rely on the (complete) Boolean algebra structure of powersets for propositional connectives and on Knaster-Tarski Fixpoint Theorem for fixpoints $\mu$ and $\nu$. The interpretations of $\nu^{\mathtt{t}}\alpha\varphi(\alpha)$ and $\mu^{\mathtt{t}}\alpha\varphi(\alpha)$ (for $\mathtt{t}$ closed) are defined to be the interpretations resp. of $\varphi^{[\![\mathtt{t}]\!]}(\top)$ and $\varphi^{[\![\mathtt{t}]\!]}(\bot)$, where e.g. $\varphi^0(\top) := \top$ and $\varphi^{n+1}(\top) := \varphi(\varphi^n(\top))$. We give the cases of the atomic modalities in Fig. 12 (where for simplicity we assume formulae to be closed). It can be checked that, when restricting to polynomial types, one gets the coalgebraic semantics of [30] (with sums as in [31]) extended to fixpoints.

**Internal Semantics of Formulae.** We would like to have adequacy w.r.t. the external semantics of formulae, namely that given $M : \{A \mid \varphi\}$, the global section $[\![M]\!] \in \boldsymbol{\Gamma}[\![A]\!]$ satisfies $\{\!|\varphi|\!\} \in \mathcal{P}(\boldsymbol{\Gamma}[\![A]\!])$ in the sense that $[\![M]\!] \in \{\!|\varphi|\!\}$. But in general we can only have adequacy w.r.t. an *internal* semantics $[\![\varphi]\!] \in \mathrm{Sub}([\![A]\!])$

of formulae $\varphi : A$. We sketch it here. First, $\mathrm{Sub}(X)$ is the (complete) Heyting algebra of *subobjects* of an object $X$ of $\mathcal{S}$. Explicitly, we have $S = (S(n))_n \in \mathrm{Sub}(X)$ iff for all $n > 0$, $S(n) \subseteq X(n)$ and $r^X_n(t) \in S(n)$ whenever $t \in S(n+1)$. For propositional connectives and fixpoints, the internal $[\![-]\!]$ is defined similarly as the external $\{\!|-|\!\}$, but using (complete) Heyting algebras of subobjects rather than (complete) Boolean algebras of subsets.

As for modalities, let $[\triangle]$ be of the form $[\pi_i]$, $[\mathsf{in}_i]$, $[\mathsf{next}]$ or $[\mathsf{fold}]$, and assume $[\triangle]\varphi : B$ whenever $\varphi : A$. Standard topos theoretic constructions give posets morphisms $[\![[\triangle]]\!] : \mathrm{Sub}([\![A]\!]) \to \mathrm{Sub}([\![B]\!])$ such that $[\![[\pi_i]]\!]$, $[\![[\mathsf{fold}]]\!]$ are maps of Heyting algebras, $[\![[\mathsf{in}_i]]\!]$ preserves $\vee, \bot$ and $\wedge$, while $[\![[\mathsf{next}]]\!]$ preserves $\wedge, \top$ and $\vee$. With $[\![[\triangle]\varphi]\!] := [\![[\triangle]]\!]([\![\varphi]\!])$, all the axioms and rules of Table 2 are validated for these modalities. To handle guarded recursion, it is crucial to have $[\![[\mathsf{next}]\varphi]\!] := \blacktriangleright([\![\varphi]\!])$, with $[\![[\mathsf{next}]\varphi]\!]$ true at time 1, independently from $\varphi$. As a consequence, $[\mathsf{next}]$ and $\bigcirc$ do not validate axiom $(\mathsf{P})$ (Table 2), and $\Diamond[\mathsf{hd}]\varphi$ can "lie" about the next time step. We let $[\![[\mathsf{box}]\varphi]\!] := \boldsymbol{\Delta}(\{\!|\varphi|\!\})$.

The modality $[\mathsf{ev}(\psi)]$ is a bit more complex. For $\psi : B$ and $\varphi : A$, the formula $[\mathsf{ev}(\psi)]\varphi$ is interpreted as a *logical predicate* in the sense of [29, §9.2 & Prop. 9.2.4]. The idea is that for a term $M : \{B \to A \mid [\mathsf{ev}(\psi)]\varphi\}$, the global section $\mathsf{ev} \circ \langle [\![M]\!], x\rangle \in \boldsymbol{\Gamma}[\![A]\!]$ should satisfy $\varphi$ whenever $x \in \boldsymbol{\Gamma}[\![B]\!]$ satisfies $\psi$. We refer to [28, §D] for details.

Our semantics are both correct w.r.t. the full modal theories of Def. 6.2.

**Lemma 7.2.** *If* $\vdash^A_{\mathrm{c}} \varphi$ *then* $\{\!|\varphi|\!\} = \{\!|\top|\!\}$. *If* $\vdash^A \varphi$ *then* $[\![\varphi]\!] = [\![\top]\!]$.

**The Safe Fragment.** For $\alpha$ (positive and) guarded in $\varphi$, the internal semantics of $\theta\alpha\varphi$ is somewhat meaningless because $\mathcal{S}$ has *unique* guarded fixpoints [13, §2.5]. In particular, the typing $\mathsf{fix}(s).\mathsf{Cons}^{\mathsf{g}}\ a\ s : \{\mathsf{Str}^{\mathsf{g}} A \mid \Diamond[\varphi]\}$ for arbitrary $a : A$ and $\varphi : \mathsf{Str}^{\mathsf{g}} A$ (extending §2) is indeed verified by the $\mathcal{S}$ semantics $[\![-]\!]$. This prevents us from adequacy w.r.t. the external semantics in general. But this is possible for *safe* formulae since in this case we have:

**Proposition 7.3.** *If* $\varphi : A$ *is safe then* $\{\!|\varphi|\!\} = \boldsymbol{\Gamma}[\![\varphi]\!]$.

Proposition 7.3 gives the subtyping rule $\{\blacksquare A \mid [\mathsf{box}]\varphi\} \equiv \blacksquare\{A \mid \varphi\}$ (Fig. 11), which makes available the comonad structure of $\blacksquare$ on $[\mathsf{box}]\varphi$ when $\varphi$ is safe. Recall that in safe formulae, implications can only occur under a $[\mathsf{box}]$ modality and thus in *closed* subformulae. It is crucial for Prop. 7.3 that infs and sups are pointwise in the subobject lattices of $\mathcal{S}$, so that conjunctions and disjunctions are interpreted as with the usual classical Kripke semantics (see e.g. [47, §VI.7]). This does not hold for implications!

The second key to Prop. 7.3 is the following. For $L$ a complete lattice, a Scott *cocontinuous* function $L \to L$ is a Scott continuous function $L^{\mathrm{op}} \to L^{\mathrm{op}}$, *i.e.* which preserves codirected infs. For a safe $\alpha : A \vdash \varphi : A$, the poset maps $[\![\varphi]\!] : \mathrm{Sub}([\![A]\!]) \to \mathrm{Sub}([\![A]\!])$ and $\{\!|\varphi|\!\} : \mathcal{P}(\boldsymbol{\Gamma}[\![A]\!]) \to \mathcal{P}(\boldsymbol{\Gamma}[\![A]\!])$ are Scott cocontinuous. The greatest fixpoint $\nu\alpha\varphi(\alpha)$ can thus be interpreted, *both in* **Set** *and* $\mathcal{S}$, using Kleene's Fixpoint Theorem, as the infs of the interpretations of $\varphi^m(\top)$ for $m \in \mathbb{N}$. This leads to the expected coincidence of the two semantics for safe formulae.

| | | | |
|---|---|---|---|
| $x \Vdash_n \{A \mid \varphi\}$ | iff $x_n(\bullet) \in [\![\varphi]\!]^A(n)$ | $x \Vdash_n \mathsf{Fix}(X).A$ | iff $\mathsf{unfold} \circ x \Vdash_n A[\mathsf{Fix}(X).A/X]$ |
| $x \Vdash_n T_0 + T_1$ | iff $\exists i \in \{0,1\},\ \exists y \in \boldsymbol{\Gamma}[\![\lvert T_i\rvert]\!],\ x = \mathsf{in}_i \circ y$ and $y \Vdash_n T_i$ | | |
| $x \Vdash_n T_0 \times T_1$ | iff $\pi_0 \circ x \Vdash_n T_0$ and $\pi_1 \circ x \Vdash_n T_1$ | | $x \Vdash_n \mathbf{1}$ |
| $x \Vdash_n U \to T$ | iff $\forall k \leq n,\ \forall y \in \boldsymbol{\Gamma}[\![\lvert U\rvert]\!],\ y \Vdash_k U \implies \mathsf{ev} \circ \langle x, y\rangle \Vdash_k T$ | | |
| $x \Vdash_{n+1} \blacktriangleright T$ | iff $\exists y \in \boldsymbol{\Gamma}[\![\lvert T\rvert]\!],\ x = \mathsf{next} \circ y$ and $y \Vdash_n T$ | | $x \Vdash_1 \blacktriangleright T$ |
| $x \Vdash_n \blacksquare T$ | iff $\forall m > 0,\ x_n(\bullet) \Vdash_m T$ | | (where $x \in \boldsymbol{\Gamma}[\![\lvert\blacksquare T\rvert]\!]$) |
| $x \Vdash_n \forall k \cdot T$ | iff $x \Vdash_n T[\mathtt{t}/k]$ for all closed iteration terms $\mathtt{t}$ | | |

**Fig. 13.** The Realizability Semantics.

**The Smooth Fragment.** The *smooth* restriction allows for continuity properties needed to compute fixpoints iteratively, following Kleene's Fixpoint Theorem. This implies the correctness of the typing rules ($\nu$-I) and ($\mu$-E) of Fig. 11.

**Lemma 7.4.** *Given a closed smooth $\nu\alpha\varphi(\alpha) : A$ (resp. $\mu\alpha\varphi(\alpha) : A$), the function $\{\lvert\varphi\rvert\} : \mathcal{P}(\boldsymbol{\Gamma}[\![A]\!]) \to \mathcal{P}(\boldsymbol{\Gamma}[\![A]\!])$ is Scott-cocontinuous (resp. Scott-continuous). We have $\{\lvert\nu\alpha\varphi(\alpha)\rvert\} = \bigcap_{m\in\mathbb{N}} \{\lvert\varphi^m(\top)\rvert\}$ (resp. $\{\lvert\mu\alpha\varphi(\alpha)\rvert\} = \bigcup_{m\in\mathbb{N}} \{\lvert\varphi^m(\bot)\rvert\}$).*

**The Realizability Semantics.** The correctness of the type system w.r.t. its semantics in $\mathcal{S}$ is proved with a realizability relation.

**Definition 7.5 (Realizability).** *Given a type $T$ without free iteration variable, a global section $x \in \boldsymbol{\Gamma}[\![\lvert T\rvert]\!]$ and $n > 0$, we define the realizability relation $x \Vdash_n T$ by induction on lexicographicaly ordered pairs $(n, T)$ in Fig. 13.*

**Lemma 7.6.** *Given types $T, U$ without free iteration variable, if $x \Vdash_n U$ and $U \leq T$ then $x \Vdash_n T$.*

**Theorem 7.7 (Adequacy).** *If $\vdash M : T$, where $T$ has no free iteration variable, then $[\![M]\!] \Vdash_n T$ for all $n > 0$.*

By Thm. 7.7, a program $M : B \to A$ induces a set-theoretic function $\boldsymbol{\Gamma}[\![M]\!] : \boldsymbol{\Gamma}[\![B]\!] \to \boldsymbol{\Gamma}[\![A]\!]$, $x \mapsto [\![M]\!] \circ x$. When $B$ and $A$ are polynomial (e.g. streams $\mathsf{Str}^{\mathsf{g}}\,\mathtt{B}$, $\mathsf{Str}^{\mathsf{g}}\,\mathtt{A}$ with $\mathtt{B}$, $\mathtt{A}$ constant), Møgelberg's Thm. 7.1 says that $\boldsymbol{\Gamma}[\![M]\!]$ is a function on the usual final coalgebra for $B$, $A$ in **Set** (e.g. the set of usual streams over $\mathtt{B}$ and $\mathtt{A}$). Moreover, if e.g. $M : \{\mathsf{Str}\,\mathtt{B} \mid [\mathsf{box}]\psi\} \to \{\mathsf{Str}\,\mathtt{A} \mid [\mathsf{box}]\varphi\}$, then (modulo $\boldsymbol{\Gamma\Delta} \simeq \mathrm{Id}_{\mathbf{Set}}$) given a stream $x$ that satisfies $\psi$ (*i.e.* $x \in \{\lvert\psi\rvert\}$) the stream $\boldsymbol{\Gamma}[\![M]\!](x)$ satisfies $\varphi$ (*i.e.* $\boldsymbol{\Gamma}[\![M]\!](x) \in \{\lvert\varphi\rvert\}$). See §8 for examples.

## 8 Examples

We exemplified basic manipulations of our system over §3-6. We give further examples here. The functions used in our main examples are gathered in Table 3, with the following conventions. We use the infix notation $a ::^{\mathsf{g}} s$ for $\mathsf{Cons}^{\mathsf{g}}\ a\ s$ and write $[]^{\mathsf{g}}$ for the empty colist $\mathsf{Nil}^{\mathsf{g}}$. Moreover, we use some syntactic sugar for pattern matching, e.g. assuming $s : \mathsf{CoList}^{\mathsf{g}}\,A$ we write $\mathsf{case}\,s\ \mathsf{of}\ ([]^{\mathsf{g}} \mapsto N \mid x ::^{\mathsf{g}} xs \mapsto M)$ for $\mathsf{case}(\mathsf{unfold}\ s)\ \mathsf{of}\ (y.N[\langle\rangle/y] \mid y.M[\pi_0(y)/x\,,\ \pi_1(y)/xs])$. Most of the

$$\begin{array}{ll}
\textbf{append} : & \mathsf{CoList}\,A \longrightarrow \mathsf{CoList}\,A \longrightarrow \mathsf{CoList}\,A \\
:= & \lambda s.\lambda t. \\
& \quad \mathsf{box}_\iota(\mathsf{append}^{\mathsf{g}}\ (\mathsf{unbox}\ s)\ (\mathsf{unbox}\ t))
\end{array}
\qquad
\begin{array}{ll}
\textbf{sched} : & \mathsf{Res}\,A \longrightarrow \mathsf{Res}\,A \longrightarrow \mathsf{Res}\,A \\
:= & \lambda p.\lambda q. \\
& \quad \mathsf{box}_\iota(\mathsf{sched}^{\mathsf{g}}\ (\mathsf{unbox}\ p)\ (\mathsf{unbox}\ q))
\end{array}$$

$$\begin{array}{ll}
\textbf{append}^{\mathsf{g}} : & \mathsf{CoList}^{\mathsf{g}}\,A \to \mathsf{CoList}^{\mathsf{g}}\,A \to \mathsf{CoList}^{\mathsf{g}}\,A \\
:= & \mathsf{fix}(g).\lambda s.\lambda t.\mathsf{case}\ s\ \mathsf{of} \\
& \quad \mid []^{\mathsf{g}} \ \mapsto\ t \\
& \quad \mid x ::^{\mathsf{g}} xs \ \mapsto\ x ::^{\mathsf{g}} (g \circledast xs \circledast (\mathsf{next}\ t))
\end{array}
\qquad
\begin{array}{ll}
\textbf{sched}^{\mathsf{g}} : & \mathsf{Res}^{\mathsf{g}}\,A \longrightarrow \mathsf{Res}^{\mathsf{g}}\,A \longrightarrow \mathsf{Res}^{\mathsf{g}}\,A \\
:= & \mathsf{fix}(g).\lambda p.\lambda q.\ \mathsf{case}\ p\ \mathsf{of} \\
& \quad \mid \mathsf{Ret}^{\mathsf{g}}\ a \ \mapsto\ \mathsf{Ret}^{\mathsf{g}}\ a \\
& \quad \mid \mathsf{Cont}^{\mathsf{g}}\ k \ \mapsto \\
& \quad\ \ \mathsf{let}\ \ h = \lambda i.\ \mathsf{let}\ \ \langle o, t\rangle = ki \\
& \qquad\qquad\qquad\quad \mathsf{in}\ \ \langle o, g \circledast (\mathsf{next}\ q) \circledast t\rangle \\
& \quad\ \ \mathsf{in}\ \ \mathsf{Cont}^{\mathsf{g}}\ h
\end{array}$$

$$\begin{array}{rll}
\textbf{diag} & := \lambda s.\mathsf{box}_\iota\big(\mathsf{diag}^{\mathsf{g}}\ (\mathsf{unbox}\ s)\big) & : \mathsf{Str}(\mathsf{Str}\,A) \longrightarrow \mathsf{Str}\,A \\
\textbf{diag}^{\mathsf{g}} & := \mathsf{diagaux}^{\mathsf{g}}\ (\lambda x.x) & : \mathsf{Str}^{\mathsf{g}}(\mathsf{Str}\,A) \longrightarrow \mathsf{Str}^{\mathsf{g}}\,A
\end{array}$$

$$\begin{array}{rl}
\textbf{diagaux}^{\mathsf{g}} : & (\mathsf{Str}\,A \to \mathsf{Str}\,A) \longrightarrow \mathsf{Str}^{\mathsf{g}}(\mathsf{Str}\,A) \longrightarrow \mathsf{Str}^{\mathsf{g}}\,A \\
:= & \mathsf{fix}(g).\lambda t.\lambda s.\ \mathsf{Cons}^{\mathsf{g}}\ \big((\mathsf{hd} \circ t)(\mathsf{hd}^{\mathsf{g}}\ s)\big)\ \big(g \circledast \mathsf{next}(t \circ \mathsf{tl}) \circledast (\mathsf{tl}^{\mathsf{g}}\ s)\big)
\end{array}$$

$$\begin{array}{ll}
\textbf{fb} : & \mathsf{CoNat} \longrightarrow \mathsf{CoNat} \longrightarrow \mathsf{Str}\,\mathsf{Bool} \\
:= & \lambda c.\lambda m.\ \mathsf{box}_\iota(\mathsf{fb}^{\mathsf{g}}\ (\mathsf{unbox}\ c)\ (\mathsf{unbox}\ m))
\end{array}
\qquad
\begin{array}{ll}
\textbf{fb}^{\mathsf{g}} : & \mathsf{CoNat}^{\mathsf{g}} \longrightarrow \mathsf{CoNat}^{\mathsf{g}} \longrightarrow \mathsf{Str}^{\mathsf{g}}\,\mathsf{Bool} \\
:= & \mathsf{fix}(g).\lambda c.\lambda m.\ \mathsf{case}\ c\ \mathsf{of} \\
& \quad \mid \mathsf{Z}^{\mathsf{g}} \ \mapsto\ \mathsf{ff} ::^{\mathsf{g}} g \circledast (\mathsf{next}\ m) \circledast \mathsf{next}(\mathsf{S}^{\mathsf{g}}\ (\mathsf{next}\ m)) \\
& \quad \mid \mathsf{S}^{\mathsf{g}} n \ \mapsto\ \mathsf{tt} ::^{\mathsf{g}} g \circledast n \circledast (\mathsf{next}\ m)
\end{array}$$

$$\begin{array}{ll}
\textbf{extract} : & \mathsf{Rou}^{\mathsf{g}}(\mathsf{CoList}^{\mathsf{g}}\,A) \longrightarrow \mathsf{CoList}^{\mathsf{g}}\,A \\
:= & \mathsf{fix}(g).\lambda c.\ \mathsf{case}\ c\ \mathsf{of} \\
& \quad \mid \mathsf{Over}^{\mathsf{g}} \ \mapsto\ \mathsf{Nil}^{\mathsf{g}} \\
& \quad \mid \mathsf{Cont}^{\mathsf{g}} f \ \mapsto\ f g^{\circledast}
\end{array}
\qquad
\begin{array}{ll}
\textbf{unfold} : & \mathsf{Rou}^{\mathsf{g}}\,A \longrightarrow (\blacktriangleright \mathsf{Rou}^{\mathsf{g}}\,A \to \blacktriangleright A) \longrightarrow \blacktriangleright A \\
:= & \lambda c.\ \mathsf{case}\ c\ \mathsf{of} \\
& \quad \mid \mathsf{Over}^{\mathsf{g}} \ \mapsto\ \lambda k.\ k\ (\mathsf{next}\ \mathsf{Over}^{\mathsf{g}}) \\
& \quad \mid \mathsf{Cont}^{\mathsf{g}} f \ \mapsto\ \lambda k.\ \mathsf{next}(fk)
\end{array}$$

$$\textbf{bft}^{\mathsf{g}} := \lambda t.\ \mathsf{extract}\ (\mathsf{bftaux}\ t\ \mathsf{Over}^{\mathsf{g}}) : \mathsf{Tree}^{\mathsf{g}}\,A \longrightarrow \mathsf{CoList}^{\mathsf{g}}\,A$$

$$\begin{array}{rl}
\textbf{bftaux} : & \mathsf{Tree}^{\mathsf{g}}\,A \longrightarrow \mathsf{Rou}^{\mathsf{g}}(\mathsf{CoList}^{\mathsf{g}}\,A) \longrightarrow \mathsf{Rou}^{\mathsf{g}}(\mathsf{CoList}^{\mathsf{g}}\,A) \\
:= & \mathsf{fix}(g).\lambda t.\lambda c.\ \mathsf{Cont}\ \big(\lambda k.\ (\mathsf{label}^{\mathsf{g}}\ t) ::^{\mathsf{g}} \mathsf{unfold}\ c\ \big(k \circ (g \circledast (\mathsf{son}^{\mathsf{g}}_{\ell} t))^{\circledast} \circ (g \circledast (\mathsf{son}^{\mathsf{g}}_{r} t))^{\circledast}\big)\big)
\end{array}$$

**Table 3.** Code of the Examples.

functions of Table 3 are obtained from usual recursive definitions by inserting $\circledast$ and $\mathsf{next}$ at the right places. We often write $\psi \Vert\!\mapsto \varphi$ for $[\mathsf{ev}(\psi)]\varphi$. Table 4 recaps our main examples of refinement typings, all of which (for $A$, $B$, $\mathtt{O}$, $\mathtt{I}$ constant, $\mathtt{I}$ finite and $\varphi$, $\psi$ safe and smooth) can be derived syntactically for the functions of Table 3. We use intermediate typings requiring iteration terms whenever a $\Diamond$ is involved. Below, "$\boldsymbol{\Gamma}[\![M]\!]$ satisfies $\varphi$" means $\boldsymbol{\Gamma}[\![M]\!] \in \{|\varphi|\}$ (modulo $\boldsymbol{\Gamma}\boldsymbol{\Delta} \simeq \mathrm{Id}_{\mathbf{Set}}$, see §7). We refer to [28, §E] for details.

*Example 8.1 (The Append Function on CoLists).* Our system can derive that $\boldsymbol{\Gamma}[\![\mathsf{append}]\!]$ returns a non-empty colist if one of its argument is non-empty. Using $\Diamond[\mathsf{nil}]$ (which says that a colist is finite), we can derive that $\boldsymbol{\Gamma}[\![\mathsf{append}]\!]$ returns a finite colist if its arguments are both finite. This involves the intermediate typing

$$\forall k \cdot \forall \ell \cdot \big(\big\{\mathsf{CoList}^{\mathsf{g}}\,A \ \big|\ \Diamond^{k}[\mathsf{nil}]\big\} \to \big\{\mathsf{CoList}^{\mathsf{g}}\,A \ \big|\ \Diamond^{\ell}[\mathsf{nil}]\big\} \to \big\{\mathsf{CoList}^{\mathsf{g}}\,A \ \big|\ \Diamond^{k+\ell}[\mathsf{nil}]\big\}\big)$$

In addition, if the first argument of $\boldsymbol{\Gamma}[\![\mathsf{append}]\!]$ has an element which satisfies $\varphi$, then the result has an element which satisfies $\varphi$. The same holds if the first argument is finite while the second one has an element which satisfies $\varphi$ [28, §E.6]. □

**Map over coinductive streams** (with $\triangle$ either $\Box$, $\Diamond$, $\Diamond\Box$ or $\Box\Diamond$)

$$\mathsf{map} : (\{B \mid \psi\} \to \{A \mid \varphi\}) \longrightarrow \{\mathsf{Str}\,B \mid [\mathsf{box}]\triangle[\mathsf{hd}]\psi\} \longrightarrow \{\mathsf{Str}\,A \mid [\mathsf{box}]\triangle[\mathsf{hd}]\varphi\}$$

**Diagonal of coinductive streams of streams** (with $\triangle$ either $\Box$ or $\Diamond\Box$)

$$\mathsf{diag} : \{\mathsf{Str}(\mathsf{Str}\,A) \mid [\mathsf{box}]\triangle[\mathsf{hd}][\mathsf{box}]\Box[\mathsf{hd}]\varphi\} \longrightarrow \{\mathsf{Str}\,A \mid [\mathsf{box}]\triangle[\mathsf{hd}]\varphi\}$$

**A fair stream of Booleans** (adapted from [17,8])

$$\mathsf{fb} : \mathsf{CoNat} \longrightarrow \mathsf{CoNat} \longrightarrow \mathsf{Str\,Bool}$$

$$\mathsf{fb}\ 0\ 1 : \{\mathsf{Str\,Bool} \mid [\mathsf{box}]\Box\Diamond[\mathsf{hd}][\mathsf{tt}] \quad \wedge \quad [\mathsf{box}]\Box\Diamond[\mathsf{hd}][\mathsf{ff}]\}$$

**Append on guarded recursive colists**

$$\mathsf{append}^{\mathsf{g}} : \{\mathsf{CoList}^{\mathsf{g}}\,A \mid [\neg\mathsf{nil}]\} \longrightarrow \mathsf{CoList}^{\mathsf{g}}\,A \longrightarrow \{\mathsf{CoList}^{\mathsf{g}}\,A \mid [\neg\mathsf{nil}]\}$$

$$\mathsf{append}^{\mathsf{g}} : \mathsf{CoList}^{\mathsf{g}}\,A \longrightarrow \{\mathsf{CoList}^{\mathsf{g}}\,A \mid [\neg\mathsf{nil}]\} \longrightarrow \{\mathsf{CoList}^{\mathsf{g}}\,A \mid [\neg\mathsf{nil}]\}$$

**Append on coinductive colists**

$$\mathsf{append} : \{\mathsf{CoList}\,A \mid [\mathsf{box}]\Diamond[\mathsf{hd}]\varphi\} \longrightarrow \mathsf{CoList}\,A \longrightarrow \{\mathsf{CoList}\,A \mid [\mathsf{box}]\Diamond[\mathsf{hd}]\varphi\}$$

$$\mathsf{append} : \{\mathsf{CoList}\,A \mid [\mathsf{box}]\Diamond[\mathsf{nil}]\} \longrightarrow \{\mathsf{CoList}\,A \mid [\mathsf{box}]\Diamond[\mathsf{hd}]\varphi\} \longrightarrow \{\mathsf{CoList}\,A \mid [\mathsf{box}]\Diamond[\mathsf{hd}]\varphi\}$$

$$\mathsf{append} : \{\mathsf{CoList}\,A \mid [\mathsf{box}]\Diamond[\mathsf{nil}]\} \longrightarrow \{\mathsf{CoList}\,A \mid [\mathsf{box}]\Diamond[\mathsf{nil}]\} \longrightarrow \{\mathsf{CoList}\,A \mid [\mathsf{box}]\Diamond[\mathsf{nil}]\}$$

**Breadth-first tree traversal**

$$\mathsf{bft}^{\mathsf{g}} : \{\mathsf{Tree}^{\mathsf{g}}\,C \mid \forall\Box[\mathsf{lbl}]\vartheta\} \longrightarrow \{\mathsf{CoList}^{\mathsf{g}}\,C \mid \Box[\mathsf{hd}]\vartheta\}$$

(à la [35] or with Hofmann's algorithm (see e.g. [10]))

**A scheduler of resumptions** (adapted from [44])

$$\mathsf{sched} : \{\mathsf{Res}\,A \mid [\mathsf{box}]\Diamond[\mathsf{Ret}]\} \longrightarrow \{\mathsf{Res}\,A \mid [\mathsf{box}]\Diamond[\mathsf{Ret}]\} \longrightarrow \{\mathsf{Res}\,A \mid [\mathsf{box}]\Diamond[\mathsf{Ret}]\}$$

$$\mathsf{sched} : \{\mathsf{Res}\,A \mid [\mathsf{box}]\Diamond[\mathsf{now}]\psi\} \longrightarrow \{\mathsf{Res}\,A \mid [\mathsf{box}]\Diamond[\mathsf{now}]\psi\} \longrightarrow \{\mathsf{Res}\,A \mid [\mathsf{box}]\Diamond[\mathsf{now}]\psi\}$$

$$\mathsf{sched} : \{\mathsf{Res}\,A \mid [\mathsf{box}]\Box\Diamond[\mathsf{Ret}]\} \longrightarrow \{\mathsf{Res}\,A \mid [\mathsf{box}]\Box\Diamond[\mathsf{Ret}]\} \longrightarrow \{\mathsf{Res}\,A \mid [\mathsf{box}]\Box\Diamond[\mathsf{Ret}]\}$$

$$\mathsf{sched} : \{\mathsf{Res}\,A \mid [\mathsf{box}]\Box\Diamond[\mathsf{out}]\vartheta\} \longrightarrow \{\mathsf{Res}\,A \mid [\mathsf{box}]\Box\Diamond[\mathsf{out}]\vartheta\} \longrightarrow \{\mathsf{Res}\,A \mid [\mathsf{box}]\Box\Diamond[\mathsf{out}]\vartheta\}$$

(where $\Diamond$ is either $\forall\Diamond$ or $\exists\Diamond$, $\Box$ is either $\forall\Box$ or $\exists\Box$, and $[\mathsf{out}]$ is either $[\wedge\mathsf{out}]$ or $[\vee\mathsf{out}]$)

**Table 4.** Some Refinement Typings (functions defined in Table 3).

*Example 8.2 (The Map Function on Streams).* The composite modalities $\Box\Diamond$ and $\Diamond\Box$ over streams are read resp. as "infinitely often" and "eventually always". Provided with a function $f : \boldsymbol{\Gamma}[\![B]\!] \to \boldsymbol{\Gamma}[\![A]\!]$ taking $b \in \boldsymbol{\Gamma}[\![B]\!]$ satisfying $\psi$ to $f(b) \in \boldsymbol{\Gamma}[\![B]\!]$ satisfying $\varphi$, the function $\boldsymbol{\Gamma}[\![\mathsf{map}]\!]$ on set-theoretic streams returns a stream which infinitely often (resp. eventually always) satisfies $\varphi$ if its stream argument infinitely often (resp. eventually always) satisfies $\psi$ [28, §E.3]. $\Box$

*Example 8.3 (The Diagonal Function).* Consider a stream of streams $s$. We have $s = (s_i \mid i \geq 0)$ where each $s_i$ is itself a stream $s_i = (s_{i,j} \mid j \geq 0)$. The *diagonal* of $s$ is then the stream $(s_{i,i} \mid i \geq 0)$. Note that $s_{i,i} = \mathsf{hd}(\mathsf{tl}^i(\mathsf{hd}(\mathsf{tl}^i(s))))$. Indeed, $\mathsf{tl}^i(s)$ is the stream of streams $(s_k \mid k \geq i)$, so that $\mathsf{hd}(\mathsf{tl}^i(s))$ is the stream $s_i$ and $\mathsf{tl}^i(\mathsf{hd}(\mathsf{tl}^i(s)))$ is the stream $(s_{i,k} \mid k \geq i)$. Taking its head thus gives $s_{i,i}$. In the $\mathsf{diag}$ function of Table 3, the auxiliary higher-order function $\mathsf{diagaux}^{\mathsf{g}}$ iterates the coinductive $\mathsf{tl}$ over the head of the stream of streams $s$. We write $\circ$ for function composition, so that assuming $s : \mathsf{Str}^{\mathsf{g}}(\mathsf{Str}\,A)$ and $t : \mathsf{Str}\,A \to \mathsf{Str}\,A$, we have (on the *coinductive* type $\mathsf{Str}\,A$), $(\mathsf{hd}^{\mathsf{g}}\ s) : \mathsf{Str}\,A$ and

$$(\mathsf{hd} \circ t) : \mathsf{Str}\,A \to A \qquad (\mathsf{hd} \circ t)(\mathsf{hd}^{\mathsf{g}}\ s) : A \qquad (t \circ \mathsf{tl}) : \mathsf{Str}\,A \to \mathsf{Str}\,A$$

The expected refinement types for $\mathsf{diag}$ (Table 4) say that if its argument is a stream whose component streams all satisfy $\Box\varphi$, then $\boldsymbol{\Gamma}[\![\mathsf{diag}]\!]$ returns a stream whose elements all satisfy $\varphi$. Also, if the argument of $\boldsymbol{\Gamma}[\![\mathsf{diag}]\!]$ is a stream such that eventually all its component streams satisfy $\Box\varphi$, then it returns a stream which eventually always satisfies $\varphi$. See [28, §E.4] for details. □

*Example 8.4 (A Fair Stream of Booleans).* The non-regular stream $(\mathsf{fb}\ 0\ 1)$, adapted from [17,8], is of the form $\mathsf{ff}\cdot\mathsf{tt}\cdot\mathsf{ff}\cdot\mathsf{tt}^2\cdot\mathsf{ff}\cdots\mathsf{ff}\cdot\mathsf{tt}^m\cdot\mathsf{ff}\cdot\mathsf{tt}^{m+1}\cdot\mathsf{ff}\cdots$. It thus contains infinitely many $\mathsf{tt}$'s and infinitely many $\mathsf{ff}$'s. We indeed have (see [28, §E.5] for details) $(\mathsf{fb}\ 0\ 1) : \{\mathsf{Str\,Bool} \mid [\mathsf{box}]\Box\Diamond[\mathsf{hd}][\mathsf{tt}] \quad\wedge\quad [\mathsf{box}]\Box\Diamond[\mathsf{hd}][\mathsf{ff}]\}$. □

*Example 8.5 (Resumptions).* The type of resumptions $\mathsf{Res}^{\mathsf{g}}\,A$ (see Ex. 3.2) is adapted from [44]. Its guarded constructors are

$$\begin{aligned}
\mathsf{Ret}^{\mathsf{g}} &:= \lambda a.\ \mathsf{fold}(\mathsf{in}_0\ a) : A \longrightarrow \mathsf{Res}^{\mathsf{g}}\,A\\
\mathsf{Cont}^{\mathsf{g}} &:= \lambda k.\ \mathsf{fold}(\mathsf{in}_1\ k) : (\mathtt{I} \to (\mathtt{O} \times \blacktriangleright \mathsf{Res}^{\mathsf{g}}\,A)) \longrightarrow \mathsf{Res}^{\mathsf{g}}\,A
\end{aligned}$$

$\mathsf{Ret}^{\mathsf{g}}(a)$ represents a computation which returns the value $a : A$, while $\mathsf{Cont}^{\mathsf{g}}\langle f, k\rangle$ (with $\langle f, k\rangle : \mathtt{I} \to (\mathtt{O} \times \blacktriangleright \mathsf{Res}^{\mathsf{g}}\,A))$ represents a computation which on input $\mathtt{i} : \mathtt{I}$ outputs $f\mathtt{i} : \mathtt{O}$ and continues with $k\mathtt{i} : \blacktriangleright \mathsf{Res}^{\mathsf{g}}\,A$. Given $p, q : \mathsf{Res}^{\mathsf{g}}\,A$, the scheduler $(\mathsf{sched}^{\mathsf{g}}\ p\ q)$, adapted from [44], first evaluates $p$. If $p$ returns, then the whole computation returns, with the same value. Otherwise, $p$ evaluates to say $\mathsf{Cont}^{\mathsf{g}}\langle f, k\rangle$. Then $(\mathsf{sched}^{\mathsf{g}}\ p\ q)$ produces a computation which on input $\mathtt{i} : \mathtt{I}$ outputs $f\mathtt{i}$ and continues with $(\mathsf{sched}^{\mathsf{g}}\ q\ (k\mathtt{i}))$, thus switching arguments.

Let $\mathtt{I}$ be a finite base type (so that $\mathsf{Res}^{\mathsf{g}}\,A$ is *finitary* polynomial). Let $\psi : A$, $\vartheta : \mathtt{O}$ and $\varphi : \mathsf{Res}^{\mathsf{g}}\,A$. We have the following formulae (where $\mathtt{i} : \mathtt{I}$):

$$\begin{aligned}
[\mathsf{Ret}] &:= [\mathsf{fold}][\mathsf{in}_0]\top &\qquad [\mathsf{out}_{\mathtt{i}}]\vartheta &:= [\mathsf{fold}][\mathsf{in}_1]\,([\mathtt{i}] \Vert\!\!\to [\pi_0]\vartheta)\\
[\mathsf{now}]\psi &:= [\mathsf{fold}][\mathsf{in}_0]\psi & \bigcirc_{\mathtt{i}}\varphi &:= [\mathsf{fold}][\mathsf{in}_1]\,([\mathtt{i}] \Vert\!\!\to [\pi_1][\mathsf{next}]\varphi)
\end{aligned}$$

The formula $[\mathsf{Ret}]$ (resp. $[\mathsf{now}]\psi$) holds on a resumption which immediately returns (resp. with a value satisfying $\psi$) and we have $\mathsf{Ret}^{\mathsf{g}} : A \to \{\mathsf{Res}^{\mathsf{g}}\,A \mid [\mathsf{Ret}]\}$, $\mathsf{Ret}^{\mathsf{g}} : \{A \mid \psi\} \to \{\mathsf{Res}^{\mathsf{g}}\,A \mid [\mathsf{now}]\psi\}$. Moreover, the typings

$$\begin{aligned}
\mathsf{Cont}^{\mathsf{g}} &: \{\mathtt{I} \to (\mathtt{O} \times \blacktriangleright \mathsf{Res}^{\mathsf{g}}\,A) \mid [\mathtt{i}] \Vert\!\!\to [\pi_0]\vartheta\} &&\longrightarrow \{\mathsf{Res}^{\mathsf{g}}\,A \mid [\mathsf{out}_{\mathtt{i}}]\vartheta\}\\
\mathsf{Cont}^{\mathsf{g}} &: \{\mathtt{I} \to (\mathtt{O} \times \blacktriangleright \mathsf{Res}^{\mathsf{g}}\,A) \mid [\mathtt{i}] \Vert\!\!\to [\pi_1][\mathsf{next}]\varphi\} &&\longrightarrow \{\mathsf{Res}^{\mathsf{g}}\,A \mid \bigcirc_{\mathtt{i}}\varphi\}
\end{aligned}$$

express that $[\mathsf{out}_{\mathtt{i}}]\vartheta : \mathsf{Res}^{\mathsf{g}}\,A$ is satisfied by $\mathsf{Cont}^{\mathsf{g}}\langle f, k\rangle$ if $f\mathtt{i}$ satisfies $\vartheta$, and that $\bigcirc_{\mathtt{i}}\varphi : \mathsf{Res}^{\mathsf{g}}\,A$ is satisfied by $\mathsf{Cont}^{\mathsf{g}}\langle f, k\rangle$ if $k\mathtt{i}$ satisfies $[\mathsf{next}]\varphi$. Since $\mathtt{I}$ is a finite base type, it is possible to quantify over its inhabitants. We thus obtain $\mathsf{CTL}$-like variants of $\Box$ and $\Diamond$ (Ex. 4.3.(b) and Ex. 6.3). Namely:

$$\begin{aligned}
[\wedge\mathsf{out}]\vartheta &:= \textstyle\bigwedge_{\mathtt{i}\in\mathtt{I}}[\mathsf{out}_{\mathtt{i}}]\vartheta : \mathsf{Res}^{\mathsf{g}}\,A &\qquad \circledwedge\,\varphi &:= \textstyle\bigwedge_{\mathtt{i}\in\mathtt{I}} \bigcirc_{\mathtt{i}}\,\varphi \quad : \mathsf{Res}^{\mathsf{g}}\,A\\
[\vee\mathsf{out}]\vartheta &:= \textstyle\bigvee_{\mathtt{i}\in\mathtt{I}}[\mathsf{out}_{\mathtt{i}}]\vartheta : \mathsf{Res}^{\mathsf{g}}\,A & \circledvee\,\varphi &:= \textstyle\bigvee_{\mathtt{i}\in\mathtt{I}} \bigcirc_{\mathtt{i}}\,\varphi \quad : \mathsf{Res}^{\mathsf{g}}\,\varphi\\
\forall\Box\varphi &:= \nu\alpha.\ \varphi \wedge \circledwedge\,\alpha : \mathsf{Res}^{\mathsf{g}}\,A & \forall\Diamond\varphi &:= \mu\alpha.\ \varphi \vee \circledwedge\,\alpha : \mathsf{Res}^{\mathsf{g}}\,A\\
\exists\Box\varphi &:= \nu\alpha.\ \varphi \wedge \circledvee\,\alpha : \mathsf{Res}^{\mathsf{g}}\,A & \exists\Diamond\varphi &:= \mu\alpha.\ \varphi \vee \circledvee\,\alpha : \mathsf{Res}^{\mathsf{g}}\,A
\end{aligned}$$

Our system can prove that $\boldsymbol{\Gamma}[\![\mathsf{sched}]\!]$ returns in finite time when so do its arguments, either along *some* or along *any* sequence of inputs. We moreover have expected $\Box\Diamond$ properties for all possible (consistent) combinations of $\exists/\forall$ and $[\mathsf{Ret}]/[\vee\mathsf{out}]/[\wedge\mathsf{out}]$ (Table 4, with $\psi : A$, $\vartheta : \mathtt{O}$ safe and smooth) [28, §E.7]. □

*Example 8.6 (Breadth-First Traversal).* The function $\mathsf{bft}^{\mathsf{g}}$ of Table 3 (where $g^{\circledast}$ stands for $\lambda x.g \circledast x$) implements Martin Hofmann's algorithm for breadth-first tree traversal. This algorithm involves the higher-order type $\mathsf{Rou}^{\mathsf{g}}\, A$ (see Ex. 3.2) with constructors $\mathsf{Over}^{\mathsf{g}} := \mathsf{fold}(\mathsf{in}_0\langle\rangle) : \mathsf{Rou}^{\mathsf{g}}\, A$ and

$$\mathsf{Cont}^{\mathsf{g}} := \lambda f.\mathsf{fold}(\mathsf{in}_1 f) : \big((\blacktriangleright \mathsf{Rou}^{\mathsf{g}}\, A \to \blacktriangleright A) \to A\big) \to \mathsf{Rou}^{\mathsf{g}}\, A$$

We refer to [10] for explanations. Consider a formula $\varphi : A$. We can lift $\varphi$ to

$$[\mathsf{Rou}]\varphi := \nu\alpha.\ [\mathsf{fold}][\mathsf{in}_1]((([\mathsf{next}]\alpha \Vert\!\!\to [\mathsf{next}]\varphi) \Vert\!\!\to \varphi) : \mathsf{Rou}^{\mathsf{g}}\, A$$

We then easily derive the expected refinement type of $\mathsf{bft}^{\mathsf{g}}$ (Table 4, where $\vartheta : C$). Assume that $\vartheta$ is safe. On the one hand it is not clear what the meaning of $[\mathsf{Rou}]\vartheta$ is, because it is an unsafe formula over a non-polynomial type. On the other hand, the type of $\mathsf{bft}^{\mathsf{g}}$ in Tab. 4 has its standard expected meaning (namely: if all nodes of a tree satisfy $\vartheta$ then so do all elements of its traversal) because the types $\mathsf{Tree}^{\mathsf{g}}\, C$, $\mathsf{CoList}^{\mathsf{g}}\, C$ are polynomial and the formulae $\forall\Box[\mathsf{lbl}]\vartheta$, $\Box[\mathsf{hd}]\vartheta$ are safe. Hence, our system can prove standard statements via detours through non-standard ones, which illustrates its compositionality. We have the same typing for a usual breadth-first tree traversal with forests (*à la* [35]). See [28, §E.8]. □

### 9 Related Work

Type systems based on guarded recursion have been designed to enforce properties of programs handling coinductive types, like causality [45], productivity [5,50,18,6,25,24], or termination [62]. These properties are captured by the type systems, meaning that all well-typed programs satisfy these properties.

In an initially different line of work, temporal logics have been used as type systems for functional reactive programming (FRP), starting from LTL [32,33] to the intuitionistic modal $\mu$-calculus [17]. These works follow the Curry-Howard "proof-as-programs" paradigm, and reflect in the programming languages the constructions of the temporal logic.

The FRP approach has been adapted to guarded recursion, e.g. for the absence of space leaks [44], or the absence of time leaks, with the Fitch-style system of [7]. This more recently lead [8] to consider liveness properties with an FRP approach based on guarded recursion. In this system, the guarded $\lambda$-calculus (presented in a Fitch-style type system) is extended with a delay modality (written $\bigcirc$) together with a "until type" $A$ Until $B$. Following the Curry-Howard correspondence, $A$ Until $B$ is eliminated with a specific recursor, based on the usual unfolding of Until in LTL, and distinct from the guarded fixpoint operator.

In these Curry-Howard approaches, temporal operators are wired into the structure of types. This means that there is no separation between the program and the proof that it satisfies a given temporal property. Different type formers having different program constructs, different temporal specifications for the same program may lead to different actual code.

We have chosen a different approach, based on refinement types, with which the structure of formulae is not reflected in the structure of types. This allows for our examples to be mostly written in a usual guarded recursive fashion (see Table 3). Of course, we indeed use the modality $\blacksquare$ at the type level as a separation between safety and liveness properties. But different liveness properties (e.g. $\Diamond$, $\Diamond\Box$, $\Box\Diamond$) are uniformly handled with the same $\blacksquare$-type, which is moreover the expected one in the guarded $\lambda$-calculus [18].

Higher-order model checking (HOMC) [54,39] has been introduced to check *automatically* that higher-order recursion schemes, a simple form of higher-order programs with *finite* data-types, satisfy a $\mu$-calculus formula. Automatic verification of higher-order programs with infinite data-types (integers) has been explored for safety [40], termination [46], and more generally $\omega$-regular [51] properties. In presence of infinite datatypes, semi-automatic extensions of HOMC have recently been proposed [69]. In contrast with this paper, most HOMC approaches do not consider input-output behaviors on coalgebraic data. A notable exception is [41,23], but it does not handle higher-order functions (such as map), nor polynomial types such as $\mathsf{Str}(\mathsf{Str}\,A)$ (Ex. 8.3) or non-positive types such as $\mathsf{Rou}\,A$ (Ex. 8.6) and imposes a strong linearity constraint on pattern matching.

Event-driven approaches consider effects generating streams of events [61], which can be checked for temporal properties with algorithms based on (HO)MC [26,27], or, in presence of infinite datatypes, with refinement type systems [42,53]. Our iteration terms can be seen as oracles, as required by [42] to handle liveness properties, but we do not know if they allow for the non-regular specifications of [53]. While such approaches can handle infinite data types with good levels of automation, they do not have coinductive types nor branching time properties, such as the temporal specification of sched on resumptions (Ex. 8.5)

Along similar lines, branching was approached via non-determinism in [64], which also handles universal and existential properties on traces. This framework can handle CTL-like properties of the form $\exists/\forall$-$\Box/\Diamond$ (with our notation of Ex. 8.5), but not nested combinations of these (as e.g. $\exists\Box\forall\Diamond$ for sched in Ex. 8.5). It moreover does not handle coinductive types.

### 10 Conclusion and Future Work

We have presented a refinement type system for the guarded $\lambda$-calculus, with refinements expressing temporal properties stated as (alternation-free) $\mu$-calculus formulae. As we have seen, the system is general enough to prove precise behavioral input/output properties of coinductively-typed programs. Our main contribution is to handle liveness properties in presence of guarded recursive types. As seen in §2, this comes with inherent difficulties. In general, once guarded recursive functions are packed into coinductive ones using $\blacksquare$, the logical reasoning is made in our system directly on top of programs, following their shape, but requiring no further modification. We thus believe to have achieved some separation between programs and proofs.

We provided several examples. While they demonstrate the flexibility of our system, they also show that more abstraction would be welcomed when proving liveness properties. In addition, our system lacks expressiveness to prove e.g. liveness properties on breadth-first tree traversals.

We believe that our approach could be generalized to other programming languages with inductive or coinductive types. The key requirement are: (1) modalities in the temporal logic to navigate through the types of the languages, (2) a semantics to indicate when a program satisfies a formula of the temporal logic, which is sufficiently closed to the set-theoretic one for liveness properties to get their expected meaning, and (3) inference rules to reason over this realizability semantics.

Extensions of the guarded $\lambda$-calculus with dependent types have been explored [14,11,6,24]. It may be possible to extend our work to these systems. This would require to work in a Fitch-style presentation of the $\blacksquare$ modality, as in [7,12], since it is not known how to extend delayed substitutions to dependent types while retaining decidability of type-checking [15]. Also, it is appealing to investigate the generalization of our approach to sized types [1], in which guarded recursive types are representable [67].

We plan to investigate type checking. For instance, in a decidable fragment like the $\mu$-calculus on streams, one can check that a function of type $\{\mathsf{Str}^{\mathsf{g}}\, C \mid \Diamond\Box[\mathsf{hd}]\vartheta\} \to \{\mathsf{Str}^{\mathsf{g}}\, B \mid \Diamond\Box[\mathsf{hd}]\psi\}$ can be postcomposed with one of type $\{\mathsf{Str}^{\mathsf{g}}\, B \mid \Box\Diamond[\mathsf{hd}]\psi\} \to \{\mathsf{Str}^{\mathsf{g}}\, A \mid \Box\Diamond[\mathsf{hd}]\varphi\}$ (since $\Diamond\Box[\mathsf{hd}]\psi \Rightarrow \Box\Diamond[\mathsf{hd}]\psi$). Hence, we expect that some automation is possible for fragments of our logic. In presence of iteration terms, arithmetic extensions of the $\mu$-calculus [37,38] may provide interesting backends. An other direction is the interaction with HOMC. If (say) a stream over $A$ is representable in a suitable format, one may use HOMC to check whether it can be argument of a function expecting e.g. a stream of type $\{\mathsf{Str}^{\mathsf{g}}\, A \mid \Box\Diamond[\mathsf{hd}]\varphi\}$. This might provide automation for fragments of the guarded $\lambda$-calculus. Besides, the combination of refinement types with automatic techniques like predicate abstraction [57], abstract interpretation [34], or SMT solvers [66,65] has been particularly successful. More recently, the combination of refinement types inference with HOMC has been investigated [59].

We would like to explore temporal specification of general, effectful programs. To do so, we wish to develop the treatment of the coinductive resumptions monad [55], that provides a general framework to reason on effectful computations, as shown by interaction trees [70]. It would be interesting to study temporal specifications we could give to effectful programs encoded in this setting. To formalize reasoning on such examples, we would like to design an embedding of our system in a proof assistant like Coq.

Following [3], guarded recursion has been used to abstract the reasoning on step-indexing [4] that has been used to design Kripke Logical Relations [2] for typed higher-order effectful programming languages. Program logics for reasoning on such logical relations [19,20] uses this representation of step-indexing via guarded recursion. It is also found in Iris [36], a framework for higher-order concurrent separation logic. It would be interesting to explore the incorporation of temporal reasoning, especially liveness properties, in such logics.
